# Can statistical adjustment guided by causal inference improve the accuracy of effect estimation? A simulation and empirical research based on meta-analyses of case–control studies

**DOI:** 10.1186/s12911-020-01343-3

**Published:** 2020-12-11

**Authors:** Ruohua Yan, Tianyi Liu, Yaguang Peng, Xiaoxia Peng

**Affiliations:** 1 Center for Clinical Epidemiology and Evidence-Based Medicine, Beijing Children’s Hospital, Capital Medical University, National Center for Children’s Health, Nanlishilu 56, Xicheng District, Beijing, 100045 China; 2 Evidence Generation, Medical Affairs, AstraZenaca, Level 22, International Fortune Center, Jianguomenwai Avenue 8, Chaoyang District, Beijing, 100010 China

**Keywords:** Simulation, Confounder, Causal inference, Case–control study, Meta-analysis

## Abstract

**Background:**

Statistical adjustment is often considered to control confounding bias in observational studies, especially case–control studies. However, different adjustment strategies may affect the estimation of odds ratios (ORs), and in turn affect the results of their pooled analyses. Our study is aimed to investigate how to deal with the statistical adjustment in case–control studies to improve the validity of meta-analyses.

**Methods:**

Three types of adjustment strategies were evaluated including insufficient adjustment (not all preset confounders were adjusted), full adjustment (all confounders were adjusted under the guidance of causal inference), and improper adjustment (covariates other than confounders were adjusted). We carried out a series of Monte Carlo simulation experiments based on predesigned scenarios, and assessed the accuracy of effect estimations from meta-analyses of case–control studies by combining ORs calculated according to different adjustment strategies. Then we used the data from an empirical review to illustrate the replicability of the simulation results.

**Results:**

For all scenarios with different strength of causal relations, combining ORs that were comprehensively adjusted for confounders would get the most precise effect estimation. By contrast, combining ORs that were not sufficiently adjusted for confounders or improperly adjusted for mediators or colliders would easily introduce bias in causal interpretation, especially when the true effect of exposure on outcome was weak or none. The findings of the simulation experiments were further verified by the empirical research.

**Conclusions:**

Statistical adjustment guided by causal inference are recommended for effect estimation. Therefore, when conducting meta-analyses of case–control studies, the causal relationship formulated by exposure, outcome, and covariates should be firstly understood through a directed acyclic graph, and then reasonable original ORs could be extracted and combined by suitable methods.

## Background

Meta-analysis is a well-developed statistical methodology to synthesize results of multiple original studies [1]. Since it increases the sample size for a specific research question by combining data from different independent studies, meta-analysis enhances the accuracy of effect estimation and improves the strength of evidence [2]. The basic assumption of meta-analysis is that each included study provides an unbiased estimator, i.e., the variability of results is only attributed to random error but not systematic error [3]. Therefore, randomized controlled trial (RCT) with low risk of bias is acknowledged as a “combinable” study type for meta-analysis [4]. However, for certain conditions, especially in public health fields, RCTs may be unavailable in consideration of feasibility, ethics, or time, while observational studies or genome-wide association studies can provide supplementary information that experimental studies cannot reflect [5, 6].

During the past few decades, a growing number of meta-analyses are conducted in observational settings and genetic areas [7, 8]. Compared with RCTs, observational studies, especially case–control studies, are exposed to several potential risk of bias which may bring systematic errors in effect estimations [9]. Besides of selection bias and information bias, confounding is a kind of important bias that may distort the association between exposure and outcome. Particularly when the association strength is weak or medium, confounding may even reverse the direction of causal inference. Therefore, when conducting meta-analyses of case–control studies, all potential bias of original studies should be properly addressed [10, 11]. Selection bias and information bias can be evaluated and restricted by the Newcastle–Ottawa Scale in the process of meta-analyses [12]. Confounding bias, however, is always adjusted in the analysis phase of original studies. Logistic regression model is one of the most widely used approaches to control multiple confounders simultaneously, and odds ratio (OR) is a common estimator of causal effect.

In our previous study, we have made a secondary data analysis based on all meta-analyses of passive smoking and breast cancer in non-smoking women published from 1966 to 2016, as well as all original studies included in these meta-analyses [13]. We found an apparent inconsistency in statistical methodology among meta-analyses of case–control studies, including the selection of crude or adjusted OR for the calculation of pooled OR, and the number of covariates adjusted in original case–control studies. These inconsistencies might introduce heterogeneity of original studies and challenge the validity of meta-analysis. Although we detected these phenomena from a single case study, it is hard to draw conclusions and extrapolate to other meta-analyses of case–control studies. Furthermore, the empirical research cannot tell the true effect based on the counterfactual hypothesis, and thus cannot judge which adjustment strategy has the best precision in estimating the true effect.

Therefore, we designed this simulation study to assess the accuracy of effect estimations from meta-analyses by combining ORs of original case–control studies calculated according to different adjustment strategies. The strategies included fully adjustment of all preset confounders guided by causal inference, insufficiently adjustment of less confounders, and improperly adjustment of covariates other than confounders such as mediators or colliders. We set several scenarios and compared the performances of pooled ORs, and thereby provided recommendations on how to choose original ORs for meta-analyses under different circumstances. Then we used the data from an empirical review to give illustrations.

## Methods

### Simulation study

We carried out a series of Monte Carlo simulation experiments to create original case–control studies and their meta-analyses. The design of the simulation study is displayed in Additional file 1: Figure S1. We first simulated a target population with pre-determined exposure, outcome, and covariates (Additional file 1: Table S1) [14]. Then we randomly selected cases and controls from the population, and generated a number of case–control studies according to predesigned scenarios (Additional file 1: Table S2). We calculated series of ORs for each case–control study by adjusting for different covariates. Then we conducted meta-analyses to pool these ORs [15]. The above-mentioned process was repeated for 1000 times to obtain the empirical distribution of pooled OR [16]. The simulation assumed that all generated case–control studies were free from selection bias and information bias, and thus confounding bias was the major cause of systematic error that need to be carefully examined.

### Generation of target population

**Fig. 1:**
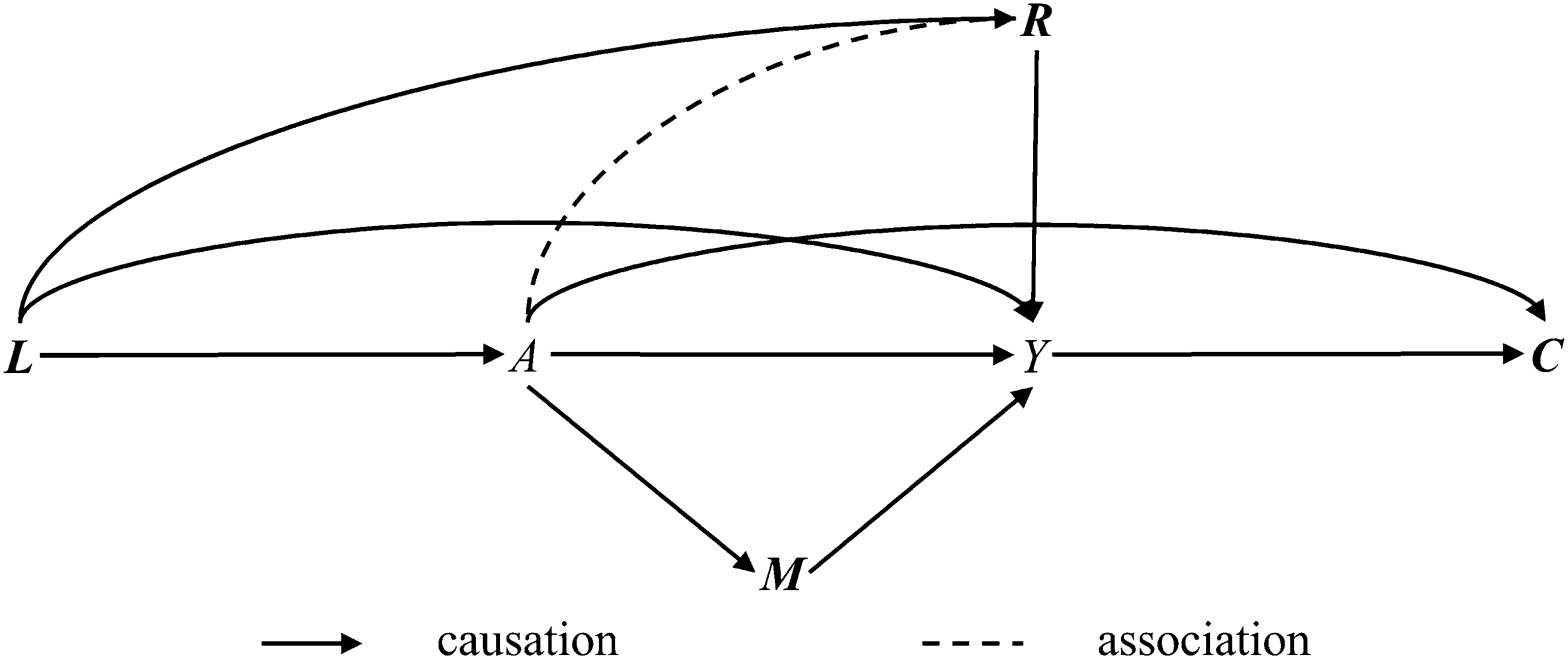
Directed acyclic graph in the target population. *A*, exposure; *Y*, outcome; ***L***, confounder; ***R***, risk factor; ***M***, mediator; ***C***, collider. The causation from *A* to *Y* can be reached in four ways: (1) the direct path *A* → *Y*; (2) the indirect path through mediators *A* → ***M*** → *Y*; the backdoor path through common causes *A* ← ***L*** → *Y* or *A* ← ***L*** → ***R*** → *Y*; and (3) the front-door path by conditioning on common effects

Suppose that we are interested in the causal effect of a dichotomous exposure variable *A* (1: exposed, 0: unexposed) on a dichotomous outcome variable *Y* (1: case, 0: control). The causation from *A* to *Y* can be reached in four ways, i.e., the direct path *A* → *Y*, the indirect path through mediators *A* → ***M*** → *Y* (***M*** denotes a set of mediators of *A* and *Y*), the backdoor path through common causes *A* ← ***L*** → *Y* or *A* ← ***L*** → ***R*** → *Y* (***L*** denotes a set of confounders of *A* and *Y*, and ***R*** denotes a set of risk factors of *Y* that have no causations with *A*), and the front-door path by conditioning on common effects  (***C*** denotes a set of colliders of *A* and *Y*). The simplified causal directed acyclic graph (DAG) between *A* and *Y* is shown in Fig. 1, and the interpretation of the DAG is provided in Additional file 1: Method S1.

Without loss of generality, we assumed a vector of 6 dichotomous confounders ***L*** = [*L*_1_, *L*_2_, …, *L*_6_] was sufficient to block all backdoor paths from *A* to *Y*. Only a dichotomous risk factor *R*, a dichotomous mediator *M*, a dichotomous collider *C* existed in the *A*-*Y* causal pathway. *R* was affected by *L*_1_, *L*_2_, *L*_3_, and *L*_4_. By specifying the positive probabilities of all variables and the association parameters, a target population with certain number of observations would be generated. Detailed method is shown in Additional file 1: Method S2.

### Generation of case–control studies

From the target population, a series of case–control studies would be generated using random sampling method. For each case–control study, 11 original ORs were calculated by different adjustment strategies. One kind of strategy was insufficient adjustment, i.e., not all confounders were controlled in the logistic regression model. The effect of *A* on *Y* was estimated by adjusting for 0 to 5 measured confounders, respectively. One kind of strategy was full adjustment, i.e., all confounders identified in Fig. 1 (*L*_1_ to *L*_6_) were controlled. The other kind of strategy was improper adjustment, i.e., covariates other than confounders were controlled in the logistic regression model. The effect of *A* on *Y* was estimated by adjusting for *L*_1_ to *L*_6_ plus *R*, *M*, *C*, and all, respectively. Detailed method is shown in Additional file 1: Method S3.

### Generation of meta-analyses

Meta-analyses were generated by combining several case–control studies, and pooled ORs were estimated under each adjustment strategy with fixed-effects model of the inverse variance method or two-stage random-effects model of the DerSimonian and Laird method as appropriate. We compared the 11 pooled ORs with the true effect specified in the target population, and thereby evaluated the performances of 11 adjustment strategies. Detailed method is shown in Additional file 1: Method S4.

### Scenario settings

Several factors may affect the performance of adjustment strategies. The first is the causation between exposure *A* and outcome *Y* in the target population, including the total effect of *A* on *Y* (OR_*AY*_), the independent associations of covariates ***U*** = [***L***, *R*, *M*, *C*] with *A* (OR_*UA*_) and *Y* (OR_*UY*_), and the correlations among different variables of ***U*** (r_*UU*_). Suppose that the positive probabilities of *A*, *Y*, and ***U*** were 20% in subjects unexposed to any parent variables, and the associations of ***U*** with *A* or *Y* were equal. We specified (1) OR_*AY*_, (2) OR_*UA*_, and (3) OR_*UY*_ as 0.2, 0.5, 0.8, 1, 1.25, 2, or 5 in different scenarios to represent strong, medium, weak, and no associations with opposite directions. We also specified (4) *r*_*UU*_ as 0, 0.2, 0.5, or 0.8, with *r*_*UU*_ ≠ 0 indicating the nonindependence of covariates.

The second factor that may affect the performance of adjustment strategies is the sample size of the original case–control studies, which involves the number of cases and the matching approach (matching ratio). To reflect various scales of original studies, we specified (5) the number of cases as 20, 100, or 500, and (6) the matching approach as frequency matching or individual matching (base on *L*_6_; 1:1, 1:2, or 1:4). Case–control studies with individual matching design should be analyzed using conditional logistic regression models.

The third factor that may affect the performance of adjustment strategies is the number of original studies included and the pooling method used in the meta-analyses. We specified (7) the number of original studies as 5, 20, or 50, referring to real meta-analyses extracted by our previous research [13]. We also specified (8) the pooling method as fixed-effects model, random-effects model, or either depended on the result of heterogeneity test (if the P value of the Q test ≥ 0.1, then fixed-effects model, else random-effects model). Detailed scenario settings are presented in Additional file 1: Table S2.

### Performance measures

A total of 32 scenarios were designed. In each scenario, 1000 meta-analyses of case–control studies were generated, and 1000 pooled ORs of exposure *A* on outcome *Y* were estimated for certain adjustment strategies. The parameter $\beta =\mathrm{ln}({\mathrm{OR}}_{AY})$ was of interest.

The repetition times (*n* = 1000) was decided by the equation $n={({Z}_{\alpha /2}\sigma /\delta )}^{2}$ [16], where ${Z}_{\alpha /2}$ was the $1-\alpha /2$ quantile of the standard normal distribution ($\alpha =0.05$), $\sigma$ was the standard deviation for $\beta$ ($\sigma =0.16$ referring to real meta-analyses extracted by our previous research [13]), and $\delta$ was the permissible difference from the true value of $\beta$ (1000 repetitions could at least ensure the accuracy of estimated $\widehat{\beta }$ achieve $\delta =0.01$, i.e., the accuracy of estimated $\widehat{\mathrm{OR}}$ archive 1%).

The Monte Carlo means of pooled ORs were calculated by $\mathrm{exp}\left(\stackrel{-}{\beta }\right)=\mathrm{exp}\left[\left(1/n\right)\sum_{i=1}^{n}{\widehat{\beta }}_{i}\right]$, while the confidence intervals (CIs) were calculated by $\mathrm{exp}\left[\overline{\beta }\pm {Z}_{\alpha /2}\times \sqrt{\left(1/n-1\right)\sum_{i=1}^{n}{\left({\widehat{\beta }}_{i}-\overline{\beta }\right)}^{2}}\right]$. The performance of different adjustment strategies was evaluated by the following 6 measures based on $\widehat{\beta }$ [16, 17]:

$$\mathrm{bias}=\frac{1}{n}\sum_{i=1}^{n}\left({\widehat{\beta }}_{i}-\beta \right)$$

$$\mathrm{relative\,bias}=\left[\frac{1}{n}\sum_{i=1}^{n}\left({\widehat{\beta }}_{i}-\beta \right)/\beta \right]\times 100\%,\mathrm{ when} \beta \ne 0$$

$$\mathrm{mean\,square\,error }\left(\mathrm{MSE}\right)=\frac{1}{n}\sum_{i=1}^{n}{\left({\widehat{\beta }}_{i}-\beta \right)}^{2}$$

$$\mathrm{width\,of\,CI}=\frac{1}{n}\sum_{i=1}^{n}\left({\widehat{\beta }}_{\mathrm{upp},i}-{\widehat{\beta }}_{\mathrm{low},i}\right)$$

$$\mathrm{coverage}=\left[\frac{1}{n}\sum_{i=1}^{n}1\left({\widehat{\beta }}_{\mathrm{low},i}\le \beta \le {\widehat{\beta }}_{\mathrm{upp},i}\right)\right]\times 100\%$$

$$\mathrm{power}=\left[ \frac{1}{n}\sum_{i=1}^{n}1\left({\widehat{\beta }}_{\mathrm{low},i}>0\mathrm{ or }{\widehat{\beta }}_{\mathrm{upp},i}<0\right)\right]\times 100\%,\mathrm{ when} \beta \ne 0$$

where ${\widehat{\beta }}_{\mathrm{upp}}$ and ${\widehat{\beta }}_{\mathrm{low}}$ represented the upper and lower limits of the CI of $\widehat{\beta }$ (based on normal distribution), respectively. Specially, when OR_*AY*_ = 1 ($\beta =0$) in scenario 1–4, coverage was equal to the probability of not making type I error, while in other scenarios that OR_*AY*_ ≠ 1 ($\beta \ne 0$), power was equal to the probability of not making type II error. To further evaluate the performance distinctions among adjustment strategies were true difference or random deviation, the Monte Carlo standard error of each measure was calculated [17]. All simulation processes and statistical analyses were conducted by SAS 9.4. The main SAS code is presented in Additional file 2.

### Empirical research

We chose an empirical meta-analysis focused on passive smoking and breast cancer in nonsmoking women to illustrate the replicability of the above simulation experiments [18]. Similar to the process of the simulation study, we firstly investigate the causal relationship among passive smoking, breast cancer, and potential confounders through a DAG. The DAG was determined on both literature evidence and subject-matter knowledge, i.e., the nodes of the DAG were identified by variables adjusted in each original case–control study, and the direction of arrow between every two nodes was judged by the author and was further approved by clinical experts.

Then we selected original ORs that were calculated by the most appropriate adjustment strategy based on the causal diagram. Fixed- or random-effects model was used to pool ORs according to the size of heterogeneity (decided by the significance of the Q test). Publication bias was assessed by funnel plots. Moreover, sensitivity analyses were conducted to combine original ORs that seemed to underestimate and overestimate the true effect, respectively, through the guidance of causal inference. All meta-analyses were performed with Review Manager 5.3.

## Results

### Effect estimations in meta-analyses of case–control studies

**Fig. 2:**
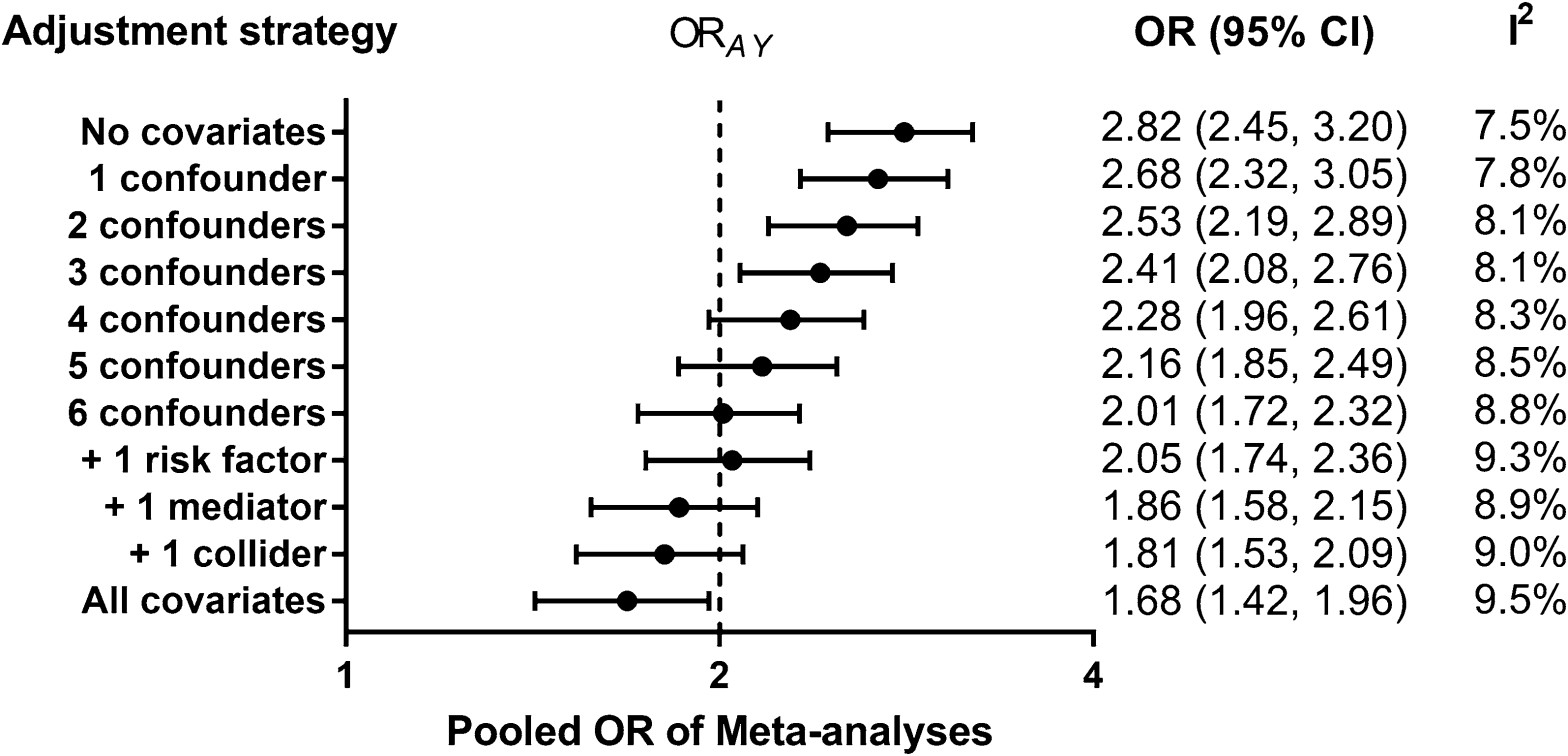
Pooled ORs of meta-analyses in scenario Ref (OR_*AY*_ = 2). OR, odds ratio; CI, confidence interval; *A*, exposure; *Y*, outcome. Pooled crude OR (no covariates) overestimated the true effect. The overestimation gradually decreased with the adjustment of more confounders. Pooled full-adjusted OR (6 confounders) had the closest effect estimation. Further adjustment of risk factor, mediator or collider slightly affected the estimation accuracy. Pooled all-adjusted OR (all covariates) underestimated the true effect

Among all scenarios defined in the simulation study, set scenario Ref be the primary analysis. Figure 2 presents the Monte Carlo pooled ORs of meta-analyses in scenario Ref. When no covariates were adjusted in original case–control studies, the average effect estimation of meta-analyses was 2.82 (95% CI 2.46–3.22), which significantly overestimated the true effect of exposure *A* on outcome *Y* (OR_*AY*_ = 2). The overestimation gradually decreased with the adjustment of more confounders. Combining original ORs that adjusted for all 6 confounders had a mean pooled OR of 2.01 (95% CI 1.72–2.32), which was the closest estimation to OR_*AY*_. Further adjusted for risk factor did not substantially change the estimation (OR 2.05; 95% CI 1.74–2.36). However, further adjusted for mediator or collider in addition to confounders did underestimate the true effect. The underestimation was similar for mediator and collider, if they had an equal association strength with exposure and outcome. A more particular interpretation of the results is shown in Additional file 1: Result S1 and Additional file 1: Figure S2.

### Performances of statistical adjustment strategies

**Fig. 3:**
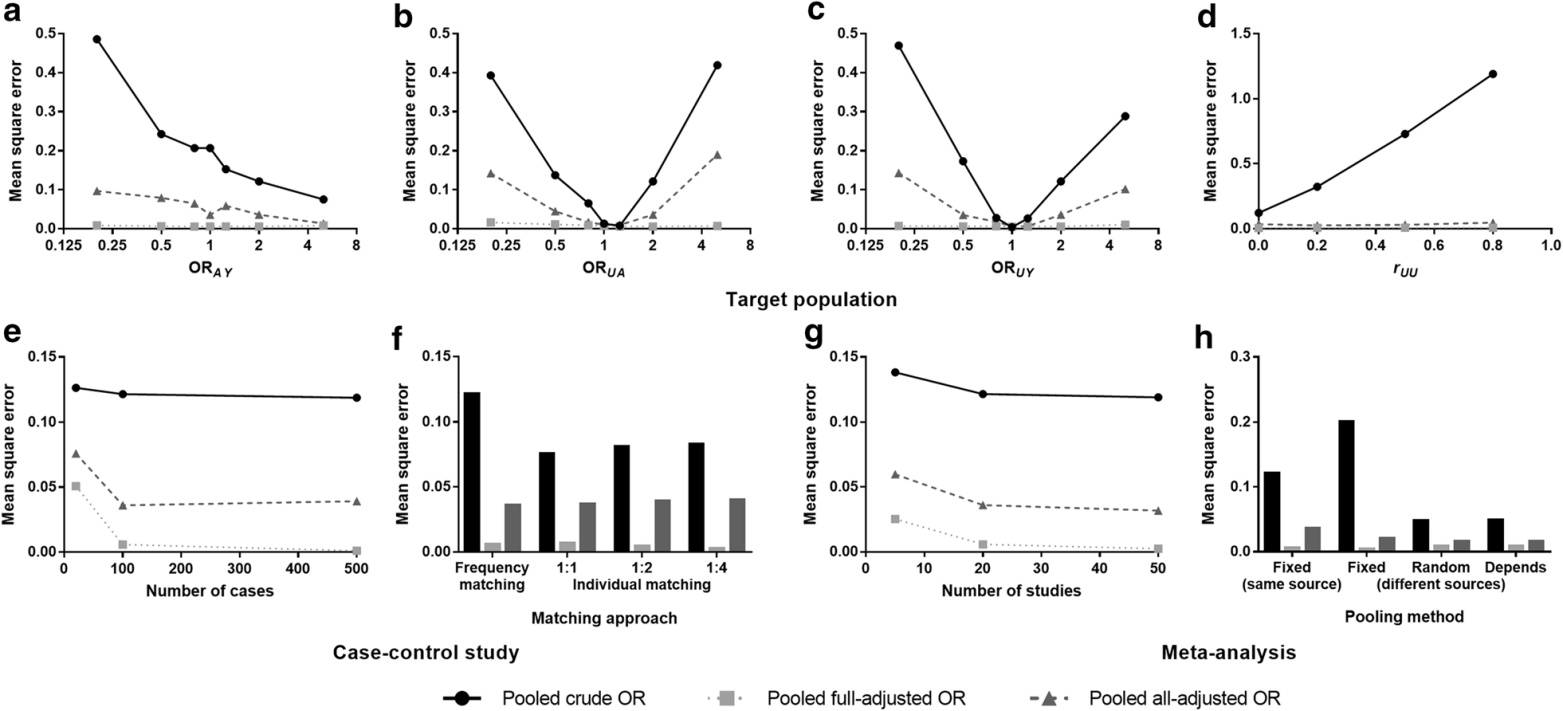
Mean square error of effect estimations under different adjustment strategies. Mean square error was presented according to different **a** OR_*AY*_, **b** OR_*UA*_, **c** OR_*UY*_, and **d** *r*_*UU*_ in the target population; **e** number of cases and **f** matching approach in the original case–control studies; and **g** number of studies and **h** pooling method in the meta-analyses. The y-axis limits differ between plots. OR, odds ratio; *A*, exposure; *Y*, outcome; ***U***, covariate. For all scenarios, pooled full-adjusted ORs showed the least mean square error. With insufficient or improper adjustment of covariates in original studies, the pooled effect estimations were away from the true value

Figure 3 displays the performances of statistical adjustment strategies in different scenarios. MSE, which is a comprehensive indicator for variance and bias ($\mathrm{MSE}=\mathrm{Var}\left(\widehat{\beta }\right)+{\mathrm{bias}}^{2}$), was closest to 0 when combining original ORs that fully adjusted for 6 confounders but not needlessly adjusted for other covariates. With more insufficient or improper adjustment of covariates in original studies, the estimated parameter $\widehat{\beta }$ was more away from the true value. Detailed data are in Additional file 1: Table S3–S10.

Coverage showed similar tendencies with MSE, i.e., CI estimations based on full adjustment strategy had the highest coverage rate to the true effect (Additional file 1: Figure S3).

**Fig. 4:**
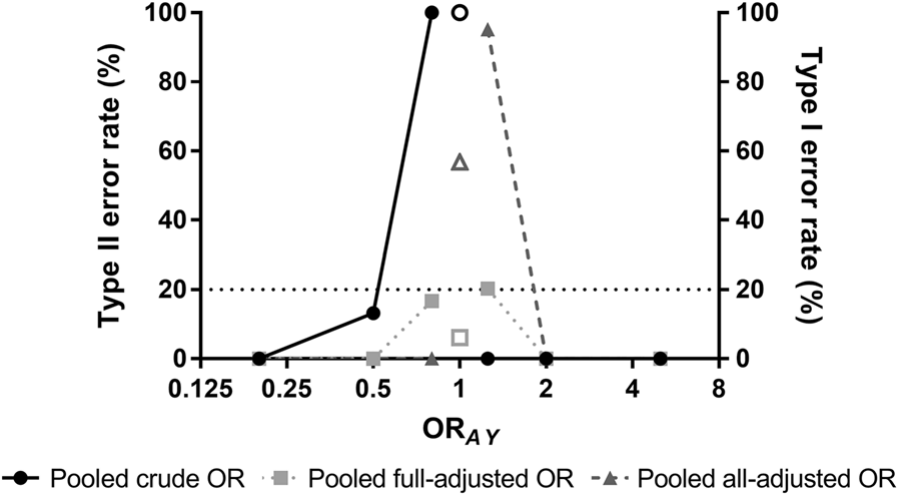
Error rate of effect estimations under different adjustment strategies. Error rate was presented according to different OR_*AY*_ in the target population. Solid symbol represents type II error (where OR_*AY*_ ≠ 1), and hollow symbol represents type I error (where OR_*AY*_ = 1). OR, odds ratio; *A*, exposure; *Y*, outcome. When OR_*AY*_ was away from 1, error rate was low for every adjustment strategy. When OR_*AY*_ was around 1, error rate was acceptable only for full adjustment strategy

Power was large in most scenarios where the total effect of exposure *A* on outcome *Y* (OR_*AY*_) was specified as 2. However, for scenarios 1–3 and 1–5 with weaker effects (OR_*AY*_ = 0.8 and 1.25, respectively), power became insufficient and type II error rate exceeded 20% under inappropriate adjustment strategies. Moreover, for scenario 1–4 with null effect (OR_*AY*_ = 1), type I error rate exceeded 5% if confounders were not adjusted under the guidance of causal inference. Error rates were acceptable only for full adjustment strategy when OR_*AY*_ was around 1 (Fig. 4).

Although the accuracy of effect estimation under each adjustment strategy was sensitive to OR_*AY*_ in the target population, it was rarely affected by OR_*UA*_, OR_*UY*_, or r_*UU*_, except for some extreme situations. Characteristics of case–control studies and meta-analyses also had little impact on the precision of pooled ORs. A more particular interpretation of the results is shown in Additional file 1: Result S2 and Additional file 1: Figure S4.

### Empirical illustrations

From the simulation experiments we noticed that, in order to get accurate estimations of causal effect in meta-analyses, ORs calculated by appropriate adjustment strategies in original case–control studies should be extracted and combined. However, how to apply our simulation results in practice is still unclear. We now use a meta-analysis conducted by Lee and Hamling in 2016 to give illustrations [18].

The meta-analysis was focused on passive smoking and risk of breast cancer in nonsmoking women. Since the research question could not be fulfilled by experimental studies due to ethical reasons, all 47 original studies involved in the meta-analysis were observational studies. Among them, 30 were case–control studies, 15 were prospective studies, and 2 were case–control studies nested within prospective studies. In the principal analysis, 29 case–control studies and 16 prospective studies were included, with the pooled effect estimations as 1.26 (95% CI 1.13–1.41) and 1.02 (95% CI 0.97–1.08), respectively. A clear difference has been found between study types (*P* < 0.001). We supposed that the result of prospective studies might be more credible, since prospective studies generally exposed to less bias and provided relatively higher quality of evidence. However, without a background knowledge of the true effect, we could not definitely conclude whether the association existed or not. Therefore, we tried to re-analyze the data from the case–control studies and give a more decisive causal inference.

First of all, the causal relationship between passive smoking and breast cancer should be understood. We summarized the information of 29 original case–control studies in Additional file 1: Table S11, where the adjusted covariates were potential confounders identified by each study. However, most studies controlled variables that showed significance in baseline comparisons or univariate analyses, without distinguishing confounders with risk factors, mediators, or colliders. We should draw a DAG to make detailed differentiation (Additional file 1: Result S3).

**Fig. 5:**
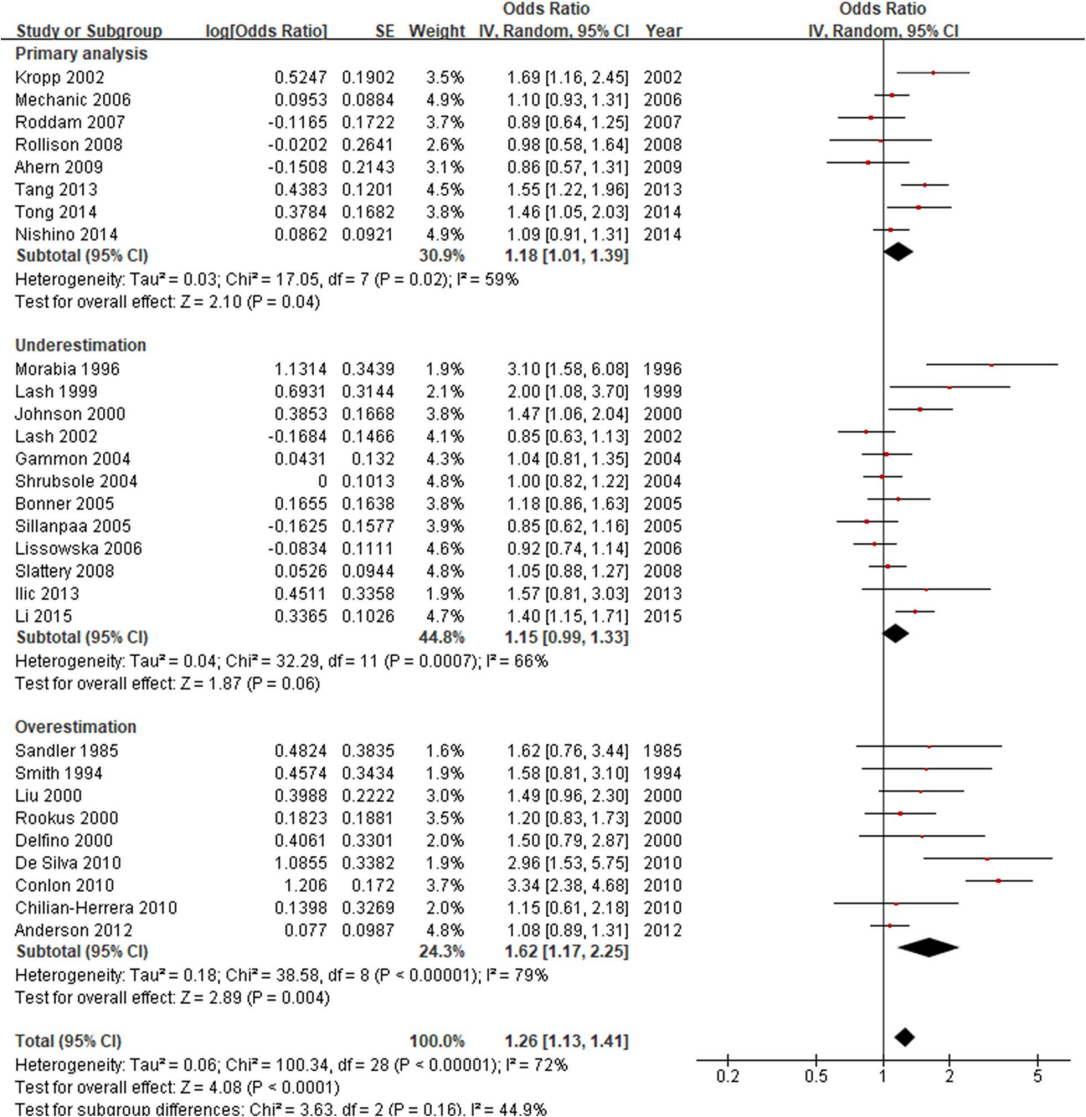
Forest plot of an empirical meta-analysis on passive smoking and breast cancer [18]. SE, standard error; IV, inverse variance; CI, confidence interval. A weak but significant association between passive smoking and breast cancer was detected in primary analysis. Underestimated results slightly shrank the effect and gave a false negative estimation, while overestimated results substantially amplified the effect

**Fig. 6:**
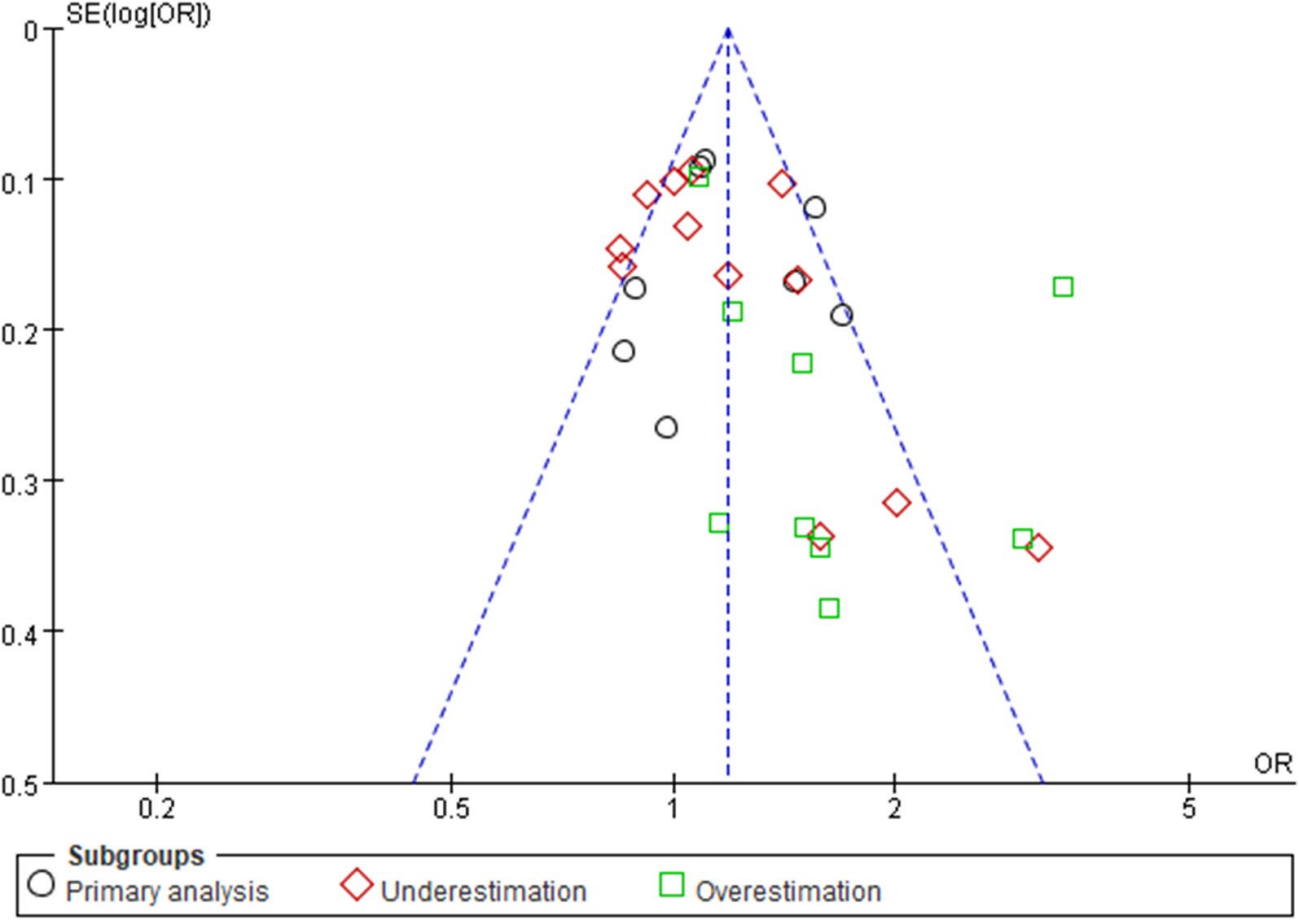
Funnel plot of an empirical meta-analysis on passive smoking and breast cancer [18]. The blue dashed lines were based on all studies included in the meta-analysis. SE, standard error; OR, odds ratio. Compared with underestimated or overestimated results that might expose to publication bias, original ORs in primary analysis were symmetrically scattered around the dashed vertical line with relatively small standard errors

Based on the DAG in Additional file 1: Figure S5, we evaluated the accuracy of original ORs and made stratification analysis. Among 29 case–control studies, 8 (27.6%) gave reasonable effect estimations and were included in the primary analysis. Meanwhile, 12 (41.4%) were underestimated due to not adjusting for negative confounders of family history (2/12), adjusting for mediators of benign breast disease (9/12), or adjusting for colliders of cardiovascular disease (1/12); 9 (31.0%) were overestimated due to not adjusting for positive confounders of age or body mass index (Additional file 1: Table S11). None of the original studies were subject to the risk of overfitting. From the forest plot in Fig. 5, we detected a weak but significant association between passive smoking and breast cancer in primary analysis (OR 1.18; 95% CI 1.01–1.39). Underestimated results slightly shrank the effect and gave a false negative estimation (OR 1.15; 95% CI 0.99–1.33). Overestimated results substantially amplified the effect (OR 1.62; 95% CI 1.17–2.25). The fixed-effects OR of primary analysis (1.18; 95% CI 1.07–1.29) was same to the random-effects OR. The funnel plot in Fig. 6 further showed that, compared with underestimated or overestimated results that might expose to publication bias (studies with few cases tended to report positive associations), original ORs in primary analysis were symmetrically scattered on both sides of 1.18 with relatively small standard errors. Therefore, we believed there is a causal relationship between passive smoking and breast cancer in non-smoking women. The conclusion was consistent with the main finding of Lee and Hamling’s review, that the relative risk from all 45 observational studies was 1.15 (95% CI 1.07–1.23) [18].

## Discussion

Our study used simulation technique and found that statistical adjustment strategy guided by causal inference would improve the accuracy of effect estimation from meta-analyses of case–control studies. For all scenarios with different strength of causal relations, combining original ORs that were comprehensively adjusted for confounders would get the most precise estimation of pooled effect, regardless of the sampling approaches of case–control studies and the scale of meta-analysis. By contrast, combining original ORs that were not sufficiently adjusted for confounders or improperly adjusted for mediators or colliders would easily introduce bias in causal interpretation, especially when the true effect of exposure on outcome was weak or none.

The findings of our simulation study were further verified by an empirical research, that is, pooled OR calculated by appropriate adjustment strategy yielded an unbiased estimation of the causal effect. By constructing a DAG with the help of adjusted variables identified by each original study in a systematic review, we could judge which study gave credible results, and combined the results together for pooled effect estimation. Other underestimated or overestimated results could be considered in sensitivity analysis to support the causal interpretation.

### Advantages and disadvantages of adjustment strategies

Our study compared three types of adjustment strategies (insufficient adjustment, full adjustment, and improper adjustment), and evaluated their impacts on pooled effect estimations of meta-analyses in different scenario settings. Full adjustment strategy is the recommended approach, because it can well eliminate the confounding bias while not cause other biases. Particularly when the effect size is weak or none, confounding bias may distort or even reverse the statistical inference. Full adjustment strategy can sufficiently control the bias in accordance with causal framework, and simultaneously avoid involving in mediation or colliding effect. The major disadvantage of full adjustment strategy is that, it is difficult to judge whether the confounders were sufficiently adjusted and whether the adjusted covariates were confounders without the help of a DAG. Therefore, the construction of DAG is very important for researchers to extract credible ORs, and should become a standard procedure of meta-analyses.

Insufficient adjustment strategy cannot completely control the confounding bias. A typical example of insufficient adjustment strategy is univariate analyses, i.e., no covariates are adjusted for the calculation of original OR. Some meta-analyses combined these crude ORs to ensure statistical homogeneity [19, 20]. However, the heterogeneity brought by confounding bias is far larger than that brought by different adjusted covariates. Since pooled crude OR does not consider any potential confounders, it shows the worst accuracy and robustness in effect estimation.

Improper adjustment strategy may be interfered by the mediation or colliding effect that reduce the estimation accuracy. But it is commonly seen in practice, because researchers of original studies tend to include as many measured variables as possible in the regression model if the sample size allows. They may not carefully distinguish confounders from mediators or colliders, which have similar association patterns with exposure and outcome. Or they may provide several ORs calculated by different models, some adjusted for clear confounders, and some adjusted for all relative factors. Meta-analyses generally pooled most-adjusted ORs, with the assumption that the more the variables are adjusted, the smaller the confounding bias [21–23]. However, if variables other than confounders are misadjusted, the pooled results will also be biased. In addition, a few meta-analyses used crude OR from part of original studies, and used adjusted OR from the other part [24, 25]. This approach cannot appropriately control the confounding bias, and cannot meet statistical homogeneity either. In summary, full adjustment is better than all other strategies for meta-analyses of case–control studies.

### Comparisons with previous studies

Nowadays, computing methods such as intelligent data analysis [26, 27], data mining [28, 29], and machine learning [30] have been increasingly used to support healthcare decision-making. Among them, simulation is becoming a powerful supplement to empirical medical researches, especially when the outcomes cannot be derived from mathematical formulae or experimental replications [31]. The thought of Monte Carlo simulation has been widely applied in the field of epidemiology [32, 33]. Regarding our study, because the true causal effect could not be obtained from real meta-analyses, and the possible adjustment strategies could not be exhausted by real case–control studies, empirical researches were not able to give confirmative conclusions, while statistical simulation was a good solution.

On the other hand, empirical research is also an important illustration of simulation results. Compared with the methodological representation, the practical application of the findings is always of value. By analyzing a motivating example, the reason for conducting the simulation study would be clarified, and the parameter settings of the scenarios would be justified. Moreover, a proper instance would help the technical paper popularize to non-technical audiences.

To our knowledge, this is the first simulation study to evaluate how adjustment strategies of original case–control studies impact the pooled effect estimations of meta-analyses. From our previous case study on meta-analyses of passive smoking and breast cancer [13], we detected an inconsistency in adjustment strategies used for calculating original ORs, which might not eliminate confounding bias but introduce new bias during meta-analyses. However, the previous study could not determine the best strategy without knowing the true effect, and could not extrapolate the best strategy to other situations as well. In the present study, by specifying the true OR of exposure on outcome and setting various parameters in multiple scenarios, we compared the accuracy of adjustment strategies under different circumstances and gave methodological recommendations correspondingly. Our findings are expected to help improve the validity of meta-analyses of case–control studies, and provide high-quality evidence for medical decision-making.

### Hypotheses and limitations

Although our findings provide important implications on how to choose original ORs of case–control studies for meta-analyses, caution is needed when the situation is more complex. First, we set a relatively fixed causal mode between exposure and outcome, and prespecified 6 confounders, a risk factor, a mediator, and a collider in the target population due to feasibility and availability considerations. While in real-world applications, the number of covariates and the inter-covariate causations were far more flexible. Since our study mainly focused on the causal attributes of the adjusted variables rather than the number of them, we made a general assumption without loss of generality. The potential impact of confounder numbers on the meta-results could be evaluated in future studies.

Second, we assumed all generated case–control studies were conducted in an ideal circumstance without selection bias and information bias. However, in practice, it is difficult to ensure the quality of original studies. Therefore, the potential biases of original studies should be carefully evaluated [12], and the consequent clinical or methodological heterogeneity among original studies should be controlled, either by statistical approaches such as random-effects model or meta-regression, or by subgroup analysis to pool all “combinable” results together [34, 35]. Otherwise, if the methodologies of original studies are far from each other, qualitative systemic reviews rather than quantitative pooled analyses are recommended [9, 36].

Third, we made all statistical adjustment in case–control studies based on logistic regression models. But in real cases, other adjustment methods such as propensity score and instrument variable are also used [37]. How to combine the results calculated by different adjustment methods need to be further investigated.

Fourth, we combined case–control studies by fixed-effects model with the inverse variance method or two-stage random-effects model with the DerSimonian and Laird method, because they are the recommended methods of the Cochrane Collaboration [38]. However, there are many other random-effects generalized linear mixed models, heterogeneity variance estimators, and CI calculation methods that might be more suitable for statistical inference in certain circumstances [39–41]. As our study did not aim at the selection of pooling methods of meta-analyses, we did not make a wider expansion. But for better practical applications, the random-effects models and the variance estimation methods should be detailly considered in the future.

Fifth, we focused on meta-analyses of case–control studies, while in most actual reviews, observational studies including case–control studies and cohort studies are combined together to calculate pooled ORs. Compared with case–control studies, cohort studies are exposed to more uncertain factors, such as different follow-up durations, different rates of loss to follow-up, etc. Whether the results of the present study are still valid for meta-analyses of cohort studies, is another important question to be answered in our future research.

## Conclusions

Statistical adjustment strategy guided by causal inference are recommended for effect estimations. Thus, when conducting meta-analyses of case–control studies, the causal relationship between exposure and outcome should be firstly understood through a DAG, and then reasonable original ORs should be extracted and combined by suitable methods to get accurate pooled ORs.

## Supplementary information

**Additional file 1:** Supplementary materials.

**Additional file 2:** Main SAS code of the simulation for scenario Ref.

## Data Availability

The datasets used and/or analysed during the current study are available from the corresponding author on reasonable request.

## Abbreviations

RCT: Randomized controlled trial
OR: Odds ratio
DAG: Directed acyclic graph
CI: Confidence interval
MSE: Mean square error
