# Supplementary material for: Can statistical adjustment guided by causal inference improve the accuracy of effect estimation? A simulation and empirical research based on meta-analyses of case–control studies
Source: BMC Med Inform Decis Mak. 2020 Dec 11;20:333. doi: 10.1186/s12911-020-01343-3 (PMC7731571; doi:10.1186/s12911-020-01343-3)
Supplement: Supplementary file 1 — Additional file 1: Supplementary materials. [file 12911_2020_1343_MOESM1_ESM.pdf]

Can statistical adjustment guided by causal inference improve the accuracy of effect estimation? A simulation and empirical research based on meta-analyses of case-control studies

Additional file 1. Supplementary materials

Ruohua Yan[1], Tianyi Liu[1,2], Yaguang Peng[1], Xiaoxia Peng[1*]

1. Center for Clinical Epidemiology and Evidence-based Medicine, Beijing Children's Hospital, Capital Medical University, National Center for Children's Health, Nanlishilu 56, Xicheng District, Beijing 100045, China

2. Evidence Generation, Medical Affairs, AstraZenaca, Level 22, International Fortune Center, Jianguomenwai Avenue 8, Chaoyang District, Beijing 100010, China

* Correspondence: pengxiaoxia@bch.com.cn

Supplementary Methods

Method S1. Interpretation of directed acyclic graph in the target population 4

Method S2. Generation of target population 5

Method S3. Generation of case-control studies 8

Method S4. Generation of meta-analyses 10

Supplementary Results

Result S1. Interpretation of effect estimations in scenario Ref 12

Result S2. Interpretation of performances of statistical adjustment strategies 13

Result S3. Construction of directed acyclic graph between passive smoking and breast cancer 15

Supplementary Tables

Table S1. The simulated distributions of exposure, outcome, and covariates in the target population of scenario Ref 17

Table S2. Scenario settings of the simulation 18

Table S3. Performances of statistical adjustment strategies in scenarios with different total effect of exposure on outcome (OR_{AY}) in target population 20

Table S4. Performances of statistical adjustment strategies in scenarios with different independent associations of covariates with exposure (OR_{UA}) in target population 24

Table S5. Performances of statistical adjustment strategies in scenarios with different independent associations of covariates with outcome (OR_{UY}) in target population 28

Table S6. Performances of statistical adjustment strategies in scenarios with different correlation coefficients among covariates (r_{UU}) in target population 32

Table S7. Performances of statistical adjustment strategies in scenarios with different number of cases (m) in original case-control studies 34

Table S8. Performances of statistical adjustment strategies in scenarios with different matching approach and matching ratio in original case-control studies 36

Table S9. Performances of statistical adjustment strategies in scenarios with different number of case-control studies (N) in meta-analyses 38

Table S10. Performances of statistical adjustment strategies in scenarios with different pooling methods in meta-analyses 40

Table S11. Case-control studies included in an empirical meta-analysis on passive smoking and breast cancer 42

Supplementary Figures

Figure S1. Flow chart of the simulation study 47

Figure S2. Pooled ORs of meta-analyses in scenario Ref' where all covariates were continuous and shared the same inter-variable relationships with scenario Ref 48

Figure S3. Coverage of effect estimations under different adjustment strategies 49

Figure S4. Directed acyclic graph in the target population of scenarios 2-4 and 3-4 50

Figure S5. Directed acyclic graph between passive smoking and breast cancer 51

References 52

Method S1. Interpretation of directed acyclic graph in the target population

Suppose that *A* is the exposure and *Y* is the outcome of the target population. The directed acyclic graph (DAG) between *A* and *Y* is shown in Figure 1. Theoretically, adjusting for ***L*** is sufficient to control confounding bias, because it blocks all backdoor paths from *A* to *Y*. However, in real case-control studies, there is no guarantee that all variables of set ***L*** are correctly measured and recorded. Even worse, analyses of practical data only yield association but not causation, and thus are hard to differentiate ***L*** (confounders), ***R*** (risk factors), ***M*** (mediators), and ***C*** (colliders), because all of them show correlations with both *A* and *Y*. Conditioning on risk factors will lower the efficiency of statistical analyses and cause inflation bias, especially when the sample sizes are small. Conditioning on mediators or colliders will incorrectly block or open the path from exposure to outcome and affect the estimation of causal effect. Hence, if researchers adjust all covariates that show significance in correlation analyses without regarding any background knowledge, the effect estimations of case-control studies will be challenged. Not to mention that the significant results are also related to the random variability, sample size, collinearity, etc., which will further increase the uncertainty. Combining these indeterminate results in a meta-analysis is apparently problematic. Therefore, when doing meta-analyses, researchers should correctly identify the "accurate" causal effects from case-control studies and pool them together by suitable methods; other "inaccurate" original ORs should be analyzed in sensitivity analysis.

Method S2. Generation of target population

Generation of target population was composed of four steps. The first step was to simulate confounder $\boldsymbol{L}$ = [L_1, L_2, …, L_6], which was the ancestor of all variables (as shown in Figure 1). By defining the positive probabilities and correlation matrix of dichotomous variables L_1 to L_6, a multivariate binomial distribution would be established through the Emrich-Piedmonte algorithm [1], and a population with 10,000 observations would be created. In detail, the multivariate binomial distribution of $\boldsymbol{L}$ was generated using a proxy multivariate normal distribution, the multivariate normal distribution was specified by a mean vector and a variance-covariance matrix, and the components of the variance-covariance matrix were determined by solving equation $\Phi\big(z(p_j), z(p_k), \rho_{jk}\big) - \delta_{jk}\sqrt{p_j q_j p_k q_k} - p_j p_k = 0$ for ρ_{jk} ($j = 1, \dots, 6,\ k = j + 1, \dots, 6$), where Φ is the cumulative distribution function for standard bivariate normal distribution, $z(\cdot)$ is the quantile function for standard normal distribution, δ_{jk} is the bivariate correlation between variables L_j and L_k (δ_{jk} was assumed to be equal to a parameter r_{UU} for all j and k; r_{UU} was specified as 0 in the reference scenario, and as 0.2, 0.5, or 0.8 in scenarios for testing different covariate correlations), and p_j and $q_j = 1 - p_j$ are the positive and negative probabilities of variable L_j, respectively (p_j was assumed to be equal to 0.2 for all j). The multivariate normal distribution was established from the above-calculated parameters and was then dichotomized to multivariate binominal distribution using the cutoff value of $z(p_j)$.

The second step was to simulate the children of $\boldsymbol{L}$, i.e., exposure A and risk factor R by logistic regression models. With the dichotomous variables L_1 to L_6 generated in

step 1, the predicted probabilities of A for 10,000 individuals would be obtained by equation $\mathrm{logit}(A_i) = \alpha_0 + \sum_{j=1}^{6} \alpha_j L_{ji}$, where i denotes the ith individual, intercept $\alpha_0 = \ln\big(\mathrm{P}(A = 1|\boldsymbol{L} = \boldsymbol{0})\big)/\ln\big(\mathrm{P}(A = 0|\boldsymbol{L} = \boldsymbol{0})\big)$ reflects the hypothetical prevalence of A in individuals unexposed to $\boldsymbol{L}$ ($\mathrm{P}(A = 1|\boldsymbol{L} = \boldsymbol{0})$ was assumed to be 0.2), and $\alpha_j = \ln\left(\mathrm{OR}_{L_jA}\right)$, $j = 1, \dots, 6$ reflect the independent associations of L_j with A (OR_{L_jA} was assumed to be equal to a parameter OR_{UA} for all j; OR_{UA} was specified as 2 in the reference scenario, and as 0.2, 0.5, 0.8, 1, 1.25, or 5 in scenarios for testing different covariate-exposure associations). Exposure A of each individual was then simulated from a Bernoulli distribution. Similarly, risk factor R would be generated based on equation $\mathrm{logit}(R_i) = \gamma_0 + \sum_{j=1}^{4} \gamma_j L_{ji}$ ($\gamma_5 = \gamma_6 = 0$ because L_5 and L_6 were assumed to have no causal effect on R), where $\gamma_0 = \ln\big(\mathrm{P}(R = 1|\boldsymbol{L} = \boldsymbol{0})\big)/\ln\big(\mathrm{P}(R = 0|\boldsymbol{L} = \boldsymbol{0})\big)$ reflects the hypothetical prevalence of R in individuals unexposed to $\boldsymbol{L}$ ($\mathrm{P}(R = 1|\boldsymbol{L} = \boldsymbol{0})$ was assumed to be 0.2), and $\gamma_j = \ln\left(\mathrm{OR}_{L_jR}\right)$, $j = 1, \dots, 4$ reflect the independent associations of $\boldsymbol{L}$ with R (OR_{L_jR} was assumed to be equal to OR_{UA} for all j).

The third step was to deal with the mediation effect from exposure A to outcome Y through mediator M. Since M was the children of A, it would be simulated according to equation $\mathrm{logit}(M_i) = \theta_0 + \theta_1 A_i$, where $\theta_0 = \ln\big(\mathrm{P}(M = 1|A = 0)\big)/\ln\big(\mathrm{P}(M = 0|A = 0)\big)$ reflects the hypothetical prevalence of M in individuals unexposed to A ($\mathrm{P}(M = 1|A = 0)$ was assumed to be 0.2), and $\theta_1 = \ln\left(\mathrm{OR}_{AM}\right)$ reflects the effect of A on M (OR_{AM} was assumed to be equal to OR_{UA}). Then outcome Y, as the children of $\boldsymbol{L}$, A, M, and R, would be simulated according to equation $\mathrm{logit}(Y_i) = \beta_0 + \sum_{j=1}^{6} \beta_j L_{ji} + \beta_7 A_i + \beta_8 M_i + \beta_9 R_i$, where $\beta_0 =$

$\ln(\mathrm{P}(Y=1|\,\boldsymbol{L}=\boldsymbol{0}, A=0, M=0, R=0))/\ln(\mathrm{P}(Y=0|\,\boldsymbol{L}=\boldsymbol{0}, A=0, M=0, R=0))$ reflects the hypothetical prevalence of Y in individuals unexposed to $\boldsymbol{L}$, A, M, and R ($\mathrm{P}(Y=1|\,\boldsymbol{L}=\boldsymbol{0}, A=0, M=0, R=0)$ was assumed to be 0.2), $\beta_j=\ln\left(\mathrm{OR}_{L_jY}\right)$, $j=1,\ldots,6$ reflect the independent associations of L_j with Y (OR_{L_jY} was assumed to be equal to a parameter OR_{UY} for all j; OR_{UY} was specified as 2 in the reference scenario, and as 0.2, 0.5, 0.8, 1, 1.25, or 5 in scenarios for testing different covariate-outcome associations), $\beta_7=\ln(\mathrm{OR}_{AY})-\mathrm{reg}_{A/M}\times\ln(\mathrm{OR}_{MY})$ (the direct effect of A on Y) and $\beta_8=\ln(\mathrm{OR}_{MY})$ (the mediation effect of M on Y) reflect the total effect of A on Y mediated by M (OR_{AY} was specified as 2 in the reference scenario, and as 0.2, 0.5, 0.8, 1, 1.25, or 5 in scenarios for testing different total effects; OR_{MY} was assumed to be equal to OR_{UY}), and $\beta_9=\ln\,(\mathrm{OR}_{RY})$ reflects the independent association of R with Y (OR_{RY} was assumed to be equal to OR_{UY}).

The last step was to simulate collider C, which was the descendent of all variables, by equation $\mathrm{logit}(C_i)=\tau_0+\tau_1 A_i+\tau_2 Y_i$, where $\tau_0=\ln(\mathrm{P}(C=1|A=0, Y=0))/\ln(\mathrm{P}(C=0|A=0, Y=0))$ reflects the hypothetical prevalence of C in individuals unexposed to A and Y ($\mathrm{P}(C=1|A=0, Y=0)$ was assumed to be 0.2), $\tau_1=\ln(\mathrm{OR}_{AC})$ reflects the effect of A on C (OR_{AC} was assumed to be equal to OR_{UA}), and $\tau_2=\ln(\mathrm{OR}_{YC})$ reflects the effect of Y on C (OR_{YC} was assumed to be equal to OR_{UY}).

Through the four-step process, a target population with 10,000 records and 11 variables would be generated. The simulated distributions of exposure, outcome, and covariates were close to what we specified (as shown in Supplementary Table S1).

Method S3. Generation of case-control studies

Observations of case-control studies were randomly sampled from the target population with pre-specified sample size and matching approach. For frequency matched studies, a same number of cases and controls were selected from the strata Y=1 and 0, respectively, using stratified random sampling method (the number of cases was specified as 100 in the reference scenario, and as 20 or 500 in scenarios for testing different sample sizes). For individual matched studies, cases were selected from the stratum Y=1 using simple random sampling method at first, and then 1, 2, or 4 controls were matched with each case by L_6.

In every case-control study, 11 original ORs were calculated according to the following adjustment strategies.

Insufficient adjustment strategies:

1) No covariates (crude OR): $\widehat{\text{OR}} = \exp(\hat{\beta}_1)$, where $\hat{\beta}_1$ is estimated from the logistic regression model $\text{logit}(Y_i) = \beta_0 + \beta_1 A_i$;

2) 1 confounder: $\widehat{\text{OR}} = \exp(\hat{\beta}_1)$, where $\hat{\beta}_1$ is estimated from the logistic regression model $\text{logit}(Y_i) = \beta_0 + \beta_1 A_i + \beta_2 L_{1i}$;

3) 2 confounders: $\widehat{\text{OR}} = \exp(\hat{\beta}_1)$, where $\hat{\beta}_1$ is estimated from the logistic regression model $\text{logit}(Y_i) = \beta_0 + \beta_1 A_i + \beta_2 L_{1i} + \beta_3 L_{2i}$;

4) 3 confounders: $\widehat{\text{OR}} = \exp(\hat{\beta}_1)$, where $\hat{\beta}_1$ is estimated from the logistic regression model $\text{logit}(Y_i) = \beta_0 + \beta_1 A_i + \beta_2 L_{1i} + \beta_3 L_{2i} + \beta_4 L_{3i}$;

5) 4 confounders: $\widehat{\text{OR}} = \exp(\hat{\beta}_1)$, where $\hat{\beta}_1$ is estimated from the logistic regression model $\text{logit}(Y_i) = \beta_0 + \beta_1 A_i + \beta_2 L_{1i} + \beta_3 L_{2i} + \beta_4 L_{3i} + \beta_5 L_{4i}$;

6) 5 confounders: $\widehat{\text{OR}} = \exp(\hat{\beta}_1)$, where $\hat{\beta}_1$ is estimated from the logistic regression model $\text{logit}(Y_i) = \beta_0 + \beta_1 A_i + \beta_2 L_{1i} + \beta_3 L_{2i} + \beta_4 L_{3i} + \beta_5 L_{4i} + \beta_6 L_{5i}$;

Full adjustment strategy:

7) 6 confounders (full-adjusted OR): $\widehat{\text{OR}} = \exp(\hat{\beta}_1)$, where $\hat{\beta}_1$ is estimated from the logistic regression model $\text{logit}(Y_i) = \beta_0 + \beta_1 A_i + \beta_2 L_{1i} + \beta_3 L_{2i} + \beta_4 L_{3i} + \beta_5 L_{4i} + \beta_6 L_{5i} + \beta_7 L_{6i}$;

Improper adjustment strategies:

8) +1 risk factor: $\widehat{\text{OR}} = \exp(\hat{\beta}_1)$, where $\hat{\beta}_1$ is estimated from the logistic regression model $\text{logit}(Y_i) = \beta_0 + \beta_1 A_i + \beta_2 L_{1i} + \beta_3 L_{2i} + \beta_4 L_{3i} + \beta_5 L_{4i} + \beta_6 L_{5i} + \beta_7 L_{6i} + \beta_8 R_i$;

9) +1 mediator: $\widehat{\text{OR}} = \exp(\hat{\beta}_1)$, where $\hat{\beta}_1$ is estimated from the logistic regression model $\text{logit}(Y_i) = \beta_0 + \beta_1 A_i + \beta_2 L_{1i} + \beta_3 L_{2i} + \beta_4 L_{3i} + \beta_5 L_{4i} + \beta_6 L_{5i} + \beta_7 L_{6i} + \beta_8 M_i$;

10) +1 collider: $\widehat{\text{OR}} = \exp(\hat{\beta}_1)$, where $\hat{\beta}_1$ is estimated from the logistic regression model $\text{logit}(Y_i) = \beta_0 + \beta_1 A_i + \beta_2 L_{1i} + \beta_3 L_{2i} + \beta_4 L_{3i} + \beta_5 L_{4i} + \beta_6 L_{5i} + \beta_7 L_{6i} + \beta_8 C_i$;

11) All covariates (all-adjusted OR): $\widehat{\text{OR}} = \exp(\hat{\beta}_1)$, where $\hat{\beta}_1$ is estimated from the logistic regression model $\text{logit}(Y_i) = \beta_0 + \beta_1 A_i + \beta_2 L_{1i} + \beta_3 L_{2i} + \beta_4 L_{3i} + \beta_5 L_{4i} + \beta_6 L_{5i} + \beta_7 L_{6i} + \beta_8 R_i + \beta_9 M_i + \beta_{10} C_i$.

Method S4. Generation of meta-analyses

Meta-analyses were generated by combining several case-control studies (the number of studies was specified as 20 in the reference scenario, and as 5 or 50 in scenarios for testing different meta scales). In the reference scenario, all original studies came from a single target population and shared a same sampling process, so that the net effect of adjustment strategies on meta-analyses could be evaluated without interference from clinical and methodological heterogeneity. In scenarios for testing different pooling methods, original studies were sampled from different sources of populations and were exposed to different sources of biases other than confounding (a systematic error of original OR that obeyed normal distribution was simulated for each case-control study). Therefore, heterogeneity might play a role in the pooled effect estimation, and should be addressed by suitable statistical models.

Fixed-effects model of the inverse variance method or two-stage random-effects model of the DerSimonian and Laird method was used for pooling ORs.

Fixed-effects model:

Let $\hat{\beta}_i = \ln(\widehat{\mathrm{OR}}_i)$, $i = 1, \ldots, N$ denotes the effect estimation of the ith case-control study, and $\mathrm{SE}(\hat{\beta}_i)$ denotes the corresponding standard error. The individual effect sizes are weighted according to the reciprocal of their variance

$$w_i = \frac{1}{\left(\mathrm{SE}(\hat{\beta}_i)\right)^2}$$

and thus the summary estimate is

$$\hat{\beta}_{IV} = \frac{\sum w_i \hat{\beta}_i}{\sum w_i} \text{ with } \mathrm{SE}(\hat{\beta}_{IV}) = \frac{1}{\sqrt{\sum w_i}}$$

The confidence interval for $\hat{\beta}_{IV}$ is estimated by $\hat{\beta}_{IV} \pm Z_{\alpha/2} \times \mathrm{SE}(\hat{\beta}_{IV})$ based on normal distribution.

The heterogeneity statistic is given by

$$Q = \sum w_i(\hat{\beta}_i - \hat{\beta})^2$$

Under the null hypothesis that there are no differences in effect estimation among original studies, Q follows a chi-squared distribution with N–1 degrees of freedom. I^2 is calculated as

$$\mathrm{I}^2 = \max\left\{0, \frac{\big(Q - (N-1)\big)}{Q} \times 100\%\right\}$$

Random-effects model:

The individual effect sizes are assumed to have a distribution $\beta_i \sim N(\beta, \sigma^2)$, where the σ^2 is estimated by

$$\hat{\sigma}^2 = \max\left\{0, \frac{Q - (N-1)}{\sum w_i - \sum w_i^2 / \sum w_i}\right\}$$

Each original effect sizes are weighted by

$$w_i' = \frac{1}{\left(\hat{\sigma}^2 + \left(\mathrm{SE}(\hat{\beta}_i)\right)^2\right)}$$

and thus the summary estimate is

$$\hat{\beta}_{DL} = \frac{\sum w_i' \hat{\beta}_i}{\sum w_i'} \text{ with } \mathrm{SE}(\hat{\beta}_{DL}) = \frac{1}{\sqrt{\sum w_i'}}$$

The confidence interval for $\hat{\beta}_{DL}$ is estimated by $\hat{\beta}_{DL} \pm Z_{\alpha/2} \times \mathrm{SE}(\hat{\beta}_{DL})$ based on normal distribution.

Result S1. Interpretation of effect estimations in scenario Ref

From Figure 2, we noticed that the insufficient adjustment strategy generally overestimated the true effect of exposure on outcome in scenario Ref, while the improper adjustment strategy usually underestimated the true effect. The direction of bias in effect estimation might be explained by the association patterns between exposure A and outcome Y. In a word, the A-Y causation could be represented by a logistic regression model $\text{logit}(Y) = \alpha + \beta A + \gamma \boldsymbol{L}$, where $\beta = \ln(\text{OR}_{AY})$, and γ reflected the effect of $\boldsymbol{L}$ on Y through the pathways of $\boldsymbol{L} \rightarrow Y$ and $\boldsymbol{L} \rightarrow \boldsymbol{R} \rightarrow Y$ (Figure 1). Because we assumed that all variables were positively correlated in scenario Ref, the values of β and γ would be greater than 0. If we didn't consider sufficient confounders $\boldsymbol{L}$ in the model, the coefficient of A would expand to fit logit(Y), and with less confounders included, $\hat{\beta}$ would be more deviated from ln(OR_{AY}). Similarly, if we considered covariates other than confounders $\boldsymbol{L}$ in the model, the coefficient of A would descend to fit logit(Y), and with more covariates included, $\hat{\beta}$ would be more deviated from ln(OR_{AY}).

Although we found some variations in effect estimations by different adjustment strategies, the causal interpretation had not been affected in this specific scenario, for the pooled ORs of meta-analyses ranged from 1.69 to 2.82, which could all be interpreted as medium effect. Similar phenomena were also observed when OR_{AY} was 0.2, 0.5, or 5 (Supplementary Table S3). Hence, when the true effect of exposure A on outcome Y was medium to strong, adjustment strategies hardly influenced the casual effect estimation. However, when the true effect was weak to none, inappropriate

adjustment strategies might distort the causal interpretation and need special attention (Supplementary Table S3). Besides, if the covariates $\boldsymbol{U} = [\boldsymbol{L}, R, M, C]$ were continuous rather than categorical, the pooled ORs also showed more sensitivity to the adjustment strategies (Supplementary Figure S2).

Result S2. Interpretation of performances of statistical adjustment strategies

Supplementary Table S3-S10 presented the performances of statistical adjustment strategies changed by various scenarios. For scenarios with different total effect of exposure A on outcome Y (OR_{AY}) in target population, the accuracy of every adjustment strategy was qualified when OR_{AY} was away from null, but the accuracy sharply decreased when OR_{AY} was around 1 (Supplementary Table S3). Only combining original ORs that fully adjusted for confounders would show precise results for weak or none effect of A on Y. Therefore, full adjustment strategy was the safest way to estimate causal effect.

For scenarios with different independent associations of covariates $\boldsymbol{U}$ with exposure A (OR_{UA}) or outcome Y (OR_{UY}), the effect estimation of each adjustment strategy was rarely affected, unless OR_{UA} or OR_{UY} was strong (Supplementary Table S4 and S5). Hence, the choice of adjustment strategies in meta-analyses of case-control studies would not be much relied on the features of covariates $\boldsymbol{U}$. We also considered 2 special scenarios where covariates $\boldsymbol{U} = [\boldsymbol{L}, R, M, C]$ had no relations to exposure A or outcome Y, and thus might not be recognized as confounders in original studies (Supplementary Figure S4). The simulated results confirmed that whether adjusted for

these variables would not affect the estimation of causal effect.

In above scenarios, we assumed all confounders $\boldsymbol{L} = [L_1, L_2, \ldots, L_6]$ were independent to each other, and full adjustment strategy was recommended for accurate effect estimation. However, if the confounders were correlated ($r_{UU} \neq 0$), less than 6 of them would be sufficient to control confounding bias, and the stronger the correlation, the fewer the variables needed to be adjusted (Supplementary Table S6). Except for unadjustment strategy that greatly overestimated the true effect when r_{UU} was strong, collinearity hardly influence the effect estimations under other adjustment strategies.

Sample sizes and matching approaches of case-control studies had little impact on the precision of pooled ORs (Supplementary Table S7 and S8). Only when the number of cases was small, sample size might not have enough power to adjust for too many covariates, and the problem of overfitting led to less stability in effect estimations. For individual matched case-control studies, crude ORs have already controlled the matching variable (L_6), and thus the accuracy was better than crude ORs of frequency matched studies.

Scales and pooling methods of meta-analyses also had little impact on the precision of pooled ORs (Supplementary Table S9 and S10). Only when the number of included studies was small, heterogeneity among studies might show up and led to less stability in effect estimations. If some included studies were obtained from different sources, random-effects model presented significantly better performance than fixed-effects model. However, neither the source of original studies nor the model used in meta-analyses affected the accuracy of full adjustment strategy. In other words, if the

causal effect was constant and all confounders were sufficiently controlled, both fixed- and random-effects model would give reasonable effect estimations. Thus, if the results of fixed- and random-effects model were parallel, the estimated pooled ORs would be trustworthy.

Result S3. Construction of directed acyclic graph between passive smoking and breast cancer

Construction of a DAG based on both literature evidence and subject-matter knowledge. We firstly identified the nodes of DAG by regarding passive smoking as exposure, breast cancer as outcome, and adjusted variables of relevant researches as covariates (Supplementary Table S11). Then we determined the direction of causal pathways from covariates to exposure or outcome. The DAG was approved by clinical experts.

To estimate the effect of passive smoking on breast cancer, women's characteristics, including demographics (e.g., age, body mass index), socioeconomics (e.g., education, occupation), and lifestyle behaviors (e.g., physical activity, alcohol intake), were adjusted in almost every case-control study. They affected passive smoking through affecting surrounding people's characteristics as well as smoking status, and affected breast cancer directly or through affecting women's reproductive factors (e.g., menarche, parity, menopause) [2]. Since women's characteristics were the ancestor variables of both passive smoking and breast cancer, they could be considered as confounders and adjusted in original studies; yet not all of them had to be adjusted

due to collinearity. Reproductive factors were also adjusted in many studies, but they were risk factors of breast cancer that had no causations with passive smoking in our hypothesis. Hence, whether adjusted for reproductive factors did not influence the causal effect estimations. Family history was clearly a cause of breast cancer [2], and it might change the smoking status of surrounding people and in turn reduce the passive smoking exposure of the woman; i.e., it was a confounder. Benign breast disease was also a cause of breast cancer [2], but it might be induced by passive smoking; i.e., it was a mediator. Except for the above-mentioned variables, a few studies also adjusted for radiation therapy (a cause of breast cancer [3, 4]), cardiovascular disease (an effect of passive smoking that showed associations with breast cancer [5]), or aspirin/nonsteroidal anti-inflammatory drug use (an indicator of high C-reactive protein that caused breast cancer and cardiovascular disease [6]). Despite the possibility of reversal causality, radiation therapy and aspirin/nonsteroidal anti-inflammatory drug use would not influence the causal pathway from passive smoking to breast cancer. Cardiovascular disease, however, might be a collider that need to be treated with more caution. For individual matched studies, cases and controls were also matched by time of recruitment and vital status to balance unmeasured confounders. The corresponding DAG is displayed in Supplementary Figure S5.

Table S1. The simulated distributions of exposure, outcome, and covariates in the target population of scenario Ref

Variables	L_1	L_2	L_3	L_4	L_5	L_6	R	M	C	A	Y
Positive probabilities	0.19*	0.20*	0.19*	0.20*	0.20*	0.20*	0.30	0.26	0.33	0.37	0.50
Correlations with covariates $\boldsymbol{U}$ (r_{UU})											
Confounder L_1	1.00	-0.01†	0.01†	0.01†	0.00†	0.00†	0.11	0.02	0.05	—	—
Confounder L_2		1.00	0.01†	-0.01†	-0.01†	-0.00†	0.14	0.02	0.04	—	—
Confounder L_3			1.00	0.02†	0.01†	-0.00†	0.13	0.02	0.06	—	—
Confounder L_4				1.00	0.01†	-0.01†	0.12	0.03	0.05	—	—
Confounder L_5					1.00	0.01†	0.00	0.01	0.04	—	—
Confounder L_6						1.00	-0.02	0.02	0.04	—	—
Risk factor R							1.00	0.02	0.05	—	—
Mediator M								1.00	0.04	—	—
Collider C									1.00	—	—
Associations with exposure A (OR_{UA})	2.03‡	2.17‡	1.97‡	1.97‡	1.99‡	2.21‡	1.02	2.07‡	2.08‡	—	—
Associations with outcome Y (OR_{UY})	2.14§	2.05§	1.94§	1.91§	1.92§	2.05§	1.99§	2.04§	2.01§	1.97§	—

OR, odds ratio; *A*, exposure; *Y*, outcome; ***U***, covariate; ***L***, confounder; *R*, risk factor; *M*, mediator; *C*, collider.

* The positive probabilities of L_1 to L_6 were specified as 20%.

† The correlations among confounders L_1 to L_6 were specified as 0 ($r_{UU} = 0$).

‡ The associations of L_1 to L_6, *M*, and *C* with *A* were specified as 2 ($\mathrm{OR}_{UA} = 2$).

§ The associations of L_1 to L_6, *R*, *M*, and *C* with *Y* were specified as 2 ($\mathrm{OR}_{UY} = 2$). ORs were calculated by multiple logistic regression models that adjusted for appropriate variables according to the assumption of causal relationships defined in Figure 1.

The simulated distributions of exposure, outcome, and covariates were close to what the pre-specification.

Table S2. Scenario settings of the simulation

Scenario	Causal relationship				Case-control studies		Meta-analyses			Description
	**OR$_{AY}$**	**OR$_{UA}$**	**OR$_{UY}$**	**r_{UU}**	**Number of cases**	**Matching approach**	**Number of original studies**	**Source of original studies**	**Model**	
**(1) Specifying the total effect of exposure A on outcome Y (OR$_{AY}$) in target population:**										
1-1	**0.2**	2	2	0	100	Frequency	20	Same	Fixed	The total effect of A on Y is
1-2	**0.5**	2	2	0	100	Frequency	20	Same	Fixed	positive (strong: 5, medium:
1-3	**0.8**	2	2	0	100	Frequency	20	Same	Fixed	2, weak: 1.25), negative
1-4	**1**	2	2	0	100	Frequency	20	Same	Fixed	(strong: 0.2, medium: 0.5,
1-5	**1.25**	2	2	0	100	Frequency	20	Same	Fixed	weak: 0.8), or none (1).
Ref	**2**	2	2	0	100	Frequency	20	Same	Fixed	
1-6	**5**	2	2	0	100	Frequency	20	Same	Fixed	
**(2) Specifying the independent associations of covariates U with exposure A (OR$_{UA}$) in target population:**										
2-1	2	**0.2**	2	0	100	Frequency	20	Same	Fixed	The independent associations
2-2	2	**0.5**	2	0	100	Frequency	20	Same	Fixed	of $\boldsymbol{L}$, M, C with A are
2-3	2	**0.8**	2	0	100	Frequency	20	Same	Fixed	positive (strong: 5, medium:
2-4	2	**1**	2	0	100	Frequency	20	Same	Fixed	2, weak: 1.25), negative
2-5	2	**1.25**	2	0	100	Frequency	20	Same	Fixed	(strong: 0.2, medium: 0.5,
Ref	2	**2**	2	0	100	Frequency	20	Same	Fixed	weak: 0.8), or none (1).
2-6	2	**5**	2	0	100	Frequency	20	Same	Fixed	
**(3) Specifying the independent associations of covariates U with outcome Y (OR$_{UY}$) in target population:**										
3-1	2	2	**0.2**	0	100	Frequency	20	Same	Fixed	The independent associations
3-2	2	2	**0.5**	0	100	Frequency	20	Same	Fixed	of $\boldsymbol{L}$, R, M, C with Y are
3-3	2	2	**0.8**	0	100	Frequency	20	Same	Fixed	positive (strong: 5, medium:
3-4	2	2	**1**	0	100	Frequency	20	Same	Fixed	2, weak: 1.25), negative
3-5	2	2	**1.25**	0	100	Frequency	20	Same	Fixed	(strong: 0.2, medium: 0.5,
Ref	2	2	**2**	0	100	Frequency	20	Same	Fixed	weak: 0.8), or none (1).
3-6	2	2	**5**	0	100	Frequency	20	Same	Fixed	
**(4) Specifying the correlation coefficients among covariates U (r_{UU}) in target population:**										

Ref	2	2	2	**0**	100	Frequency	20	Same	Fixed	The correlations among ***L*** =
4-1	2	2	2	**0.2**	100	Frequency	20	Same	Fixed	[L_1, L_2, …, L_6] are strong
4-2	2	2	2	**0.5**	100	Frequency	20	Same	Fixed	(0.8), medium (0.5), weak
4-3	2	2	2	**0.8**	100	Frequency	20	Same	Fixed	(0.2), or none (0).
**(5) Specifying the number of cases in original case-control studies:**										
5-1	2	2	2	0	**20**	Frequency	20	Same	Fixed	The scale of case-control
Ref	2	2	2	0	**100**	Frequency	20	Same	Fixed	studies is large (500),
5-2	2	2	2	0	**500**	Frequency	20	Same	Fixed	medium (100), or small (20).
**(6) Specifying the matching approach and matching ratio in original case-control studies:**										
Ref	2	2	2	0	100	**Frequency**	20	Same	Fixed	The matching approach of
6-1	2	2	2	0	100	**1:1**	20	Same	Fixed	case-control studies is
6-2	2	2	2	0	100	**1:2**	20	Same	Fixed	frequency matching or
6-3	2	2	2	0	100	**1:4**	20	Same	Fixed	individual matching (1:1, 1:2, or 1:4).
**(7) Specifying the number of case-control studies in meta-analyses:**										
7-1	2	2	2	0	100	Frequency	**5**	Same	Fixed	The number of studies
Ref	2	2	2	0	100	Frequency	**20**	Same	Fixed	included in meta-analyses is
7-2	2	2	2	0	100	Frequency	**50**	Same	Fixed	large (50), medium (20), or small (5).
**(8) Specifying the pooling methods in meta-analyses:**										
Ref	2	2	2	0	100	Frequency	20	**Same**	**Fixed**	The pooling method of meta-
8-1	2	0.2~5	0.2~5	0~0.8	20~500	Frequency or 1:1~1:4	20	**Different**	**Fixed**	analyses is fixed-effect model, random-effects
8-2	2	0.2~5	0.2~5	0~0.8	20~500	Frequency or 1:1~1:4	20	**Different**	**Random**	model, or depends on the significance of the Q test
8-3	2	0.2~5	0.2~5	0~0.8	20~500	Frequency or 1:1~1:4	20	**Different**	**Depend on Q test**	(P≥0.1: fixed, P<0.1: random).

OR, odds ratio; *A*, exposure; *Y*, outcome; ***U***, covariate; ***L***, confounder; *R*, risk factor; *M*, mediator; *C*, collider.
32 scenarios were designed for testing the performances of adjustment strategies under different causal relationships of target population, different sample sizes and matching approaches of case-control studies, and different scales and pooling methods of meta-analyses.

Table S3. Performances of statistical adjustment strategies in scenarios with different total effect of exposure on outcome (OR_{AY}) in target population

Scenario	Description	Adjustment strategy	Pooled $\widehat{OR}$ (95% $\widehat{CI}$)	I^2 (%)	Performance of $\hat{\beta} = \ln(\widehat{OR})$					
					Bias	Relative bias (%)	MSE	Width of CI	Coverage (%)	Power (%)
1-1	$OR_{AY} = 0.2$	No covariates	0.40 (0.35, 0.46)	8.2	0.69	-43.1	0.29	0.27	0	100.0
	(β = -1.6)	1 confounder	0.36 (0.32, 0.41)	8.1	0.59	-36.9	0.36	0.28	0	100.0
		2 confounders	0.33 (0.29, 0.38)	8.4	0.50	-31.2	0.26	0.29	0	100.0
		3 confounders	0.29 (0.25, 0.34)	8.3	0.38	-23.7	0.15	0.30	0	100.0
		4 confounders	0.26 (0.22, 0.30)	8.7	0.26	-15.9	0.07	0.32	12.5	100.0
		5 confounders	0.23 (0.20, 0.27)	8.7	0.15	-9.5	0.03	0.33	53.9	100.0
		6 confounders	0.21 (0.18, 0.25)	9.1	0.04	-2.7	0.01	0.34	92.5	100.0
		+ 1 risk factor	0.20 (0.17, 0.23)	9.2	-0.02	1.0	0.01	0.35	95.9	100.0
		+ 1 mediator	0.18 (0.15, 0.21)	8.7	-0.10	6.2	0.02	0.35	82.8	100.0
		+ 1 collider	0.18 (0.16, 0.22)	9.6	-0.08	4.9	0.01	0.35	86.8	100.0
		All covariates	0.15 (0.12, 0.18)	9.4	-0.30	18.5	0.10	0.38	10.4	100.0
	Monte Carlo SE (min~max)				0.0021~ 0.0029	0.13~0.18	0.0004~ 0.0033	0.0001~ 0.0002	0~0.02	0~0
1-2	$OR_{AY} = 0.5$	No covariates	0.81 (0.72, 0.93)	8.7	0.49	-70.5	0.24	0.26	0	86.8
	(β = -0.7)	1 confounder	0.76 (0.67, 0.87)	8.8	0.42	-60.7	0.18	0.27	0	98.3
		2 confounders	0.71 (0.63, 0.81)	9.3	0.36	-51.3	0.13	0.27	0	99.9
		3 confounders	0.66 (0.58, 0.75)	9.2	0.28	-39.8	0.08	0.28	2.3	100.0
		4 confounders	0.58 (0.51, 0.67)	9.4	0.16	-22.5	0.03	0.29	45.3	100.0
		5 confounders	0.54 (0.46, 0.62)	9.7	0.07	-10.2	0.01	0.30	84.9	100.0
		6 confounders	0.49 (0.42, 0.57)	9.7	-0.02	2.7	0.01	0.31	94.9	100.0
		+ 1 risk factor	0.48 (0.41, 0.56)	9.9	-0.04	5.9	0.01	0.31	92.8	100.0
		+ 1 mediator	0.44 (0.37, 0.51)	10.4	-0.14	19.7	0.03	0.32	59.2	100.0
		+ 1 collider	0.44 (0.38, 0.52)	9.9	-0.12	17.9	0.02	0.32	65.6	100.0
		All covariates	0.38 (0.32, 0.45)	11.1	-0.27	38.7	0.08	0.33	11.9	100.0
	Monte Carlo SE				0.0020~	0.30~0.39	0.0003~	0.0000~	0~0.02	0~0

	(min~max)				0.0027		0.0020	0.0002		
1-3	OR_{AY} = 0.8	No covariates	1.25 (1.11, 1.42)	8.5	0.45	-201.7	0.21	0.26	0	0
	(β = -0.2)	1 confounder	1.18 (1.04, 1.34)	8.7	0.39	-174.0	0.16	0.26	0	0
		2 confounders	1.10 (0.97, 1.26)	8.8	0.32	-144.5	0.11	0.27	0.2	0
		3 confounders	1.03 (0.90, 1.18)	9.0	0.25	-112.0	0.07	0.27	5.7	0.7
		4 confounders	0.95 (0.82, 1.08)	9.0	0.17	-74.3	0.03	0.28	35.2	12.8
		5 confounders	0.88 (0.76, 1.01)	9.0	0.09	-41.0	0.01	0.29	74.3	43.0
		6 confounders	0.80 (0.69, 0.93)	9.3	-0.00	1.3	0.01	0.30	95.6	83.3
		+ 1 risk factor	0.79 (0.68, 0.92)	9.7	-0.02	6.8	0.01	0.30	94.8	86.5
		+ 1 mediator	0.71 (0.61, 0.83)	9.5	-0.11	51.4	0.02	0.31	67.3	99.1
		+ 1 collider	0.72 (0.61, 0.83)	9.6	-0.11	50.3	0.02	0.31	69.8	99.1
		All covariates	0.63 (0.53, 0.74)	10.5	-0.24	107.5	0.06	0.32	17.8	99.9
	Monte Carlo SE				0.0020~	0.91~1.20	0.0002~	0.0000~	0~0.02	0~0.02
	(min~max)				0.0027		0.0018	0.0001		
1-4	OR_{AY} = 1	No covariates	1.57 (1.38, 1.78)	8.8	0.45	—	0.21	0.26	0	—
	(β = 0)	1 confounder	1.47 (1.29, 1.67)	9.0	0.39	—	0.15	0.26	0	—
		2 confounders	1.37 (1.20, 1.57)	9.2	0.32	—	0.11	0.27	0.4	—
		3 confounders	1.28 (1.11, 1.46)	9.6	0.24	—	0.06	0.28	6.8	—
		4 confounders	1.17 (1.02, 1.35)	10.0	0.16	—	0.03	0.28	42.0	—
		5 confounders	1.09 (0.94, 1.26)	10.3	0.09	—	0.01	0.29	76.3	—
		6 confounders	1.02 (0.88, 1.18)	10.6	0.02	—	0.01	0.30	93.9	—
		+ 1 risk factor	1.03 (0.89, 1.20)	10.8	0.03	—	0.01	0.30	93.1	—
		+ 1 mediator	0.93 (0.80, 1.08)	10.9	-0.07	—	0.01	0.30	85.2	—
		+ 1 collider	0.92 (0.79, 1.08)	10.7	-0.08	—	0.01	0.30	83.7	—
		All covariates	0.84 (0.72, 0.99)	11.4	-0.17	—	0.04	0.32	43.2	—
	Monte Carlo SE				0.0020~	—	0.0003~	0.0000~	0~0.02	—
	(min~max)				0.0026		0.0018	0.0001		
1-5	OR_{AY} = 1.25	No covariates	1.84 (1.61, 2.09)	8.5	0.39	172.7	0.15	0.26	0	100.0
	(β = 0.2)	1 confounder	1.73 (1.52, 1.98)	8.8	0.33	146.0	0.11	0.27	0.1	100.0
		2 confounders	1.64 (1.44, 1.88)	8.9	0.27	122.8	0.08	0.27	2.1	100.0
		3 confounders	1.54 (1.34, 1.77)	9.0	0.21	93.8	0.05	0.28	15.9	100.0

		4 confounders	1.44 (1.25, 1.66)	9.1	0.14	62.9	0.02	0.28	50.1	100.0
		5 confounders	1.34 (1.16, 1.55)	8.9	0.07	31.6	0.01	0.29	83.9	98.2
		6 confounders	1.24 (1.07, 1.44)	9.3	-0.01	-4.3	0.01	0.30	95.0	79.7
		+ 1 risk factor	1.23 (1.06, 1.44)	9.5	-0.01	-6.0	0.01	0.30	94.5	77.1
		+ 1 mediator	1.11 (0.96, 1.30)	9.3	-0.11	-51.4	0.02	0.31	68.9	28.7
		+ 1 collider	1.11 (0.95, 1.29)	9.6	-0.12	-53.7	0.02	0.31	65.9	26.5
		All covariates	0.99 (0.85, 1.16)	10.0	-0.23	-102.8	0.06	0.32	18.8	4.8
	Monte Carlo SE (min~max)				0.0021~ 0.0026	0.93~1.15	0.0003~ 0.0018	0.0000~ 0.0002	0~0.02	0~0.01
Ref	$OR_{AY} = 2$ ($\beta = 0.7$)	No covariates	2.82 (2.46, 3.22)	7.5	0.34	49.4	0.12	0.27	0.1	100.0
		1 confounder	2.68 (2.34, 3.07)	7.8	0.29	42.4	0.09	0.27	1.7	100.0
		2 confounders	2.53 (2.21, 2.91)	8.1	0.24	34.1	0.06	0.28	7.4	100.0
		3 confounders	2.41 (2.09, 2.78)	8.1	0.19	27.0	0.04	0.28	25.5	100.0
		4 confounders	2.28 (1.98, 2.63)	8.3	0.13	19.0	0.02	0.29	57.3	100.0
		5 confounders	2.16 (1.87, 2.50)	8.5	0.08	11.4	0.01	0.29	81.5	100.0
		6 confounders	2.01 (1.74, 2.34)	8.8	0.01	1.0	0.01	0.30	94.0	100.0
		+ 1 risk factor	2.05 (1.76, 2.38)	9.3	0.02	3.4	0.01	0.30	93.5	100.0
		+ 1 mediator	1.86 (1.59, 2.16)	8.9	-0.07	-10.8	0.01	0.31	83.7	100.0
		+ 1 collider	1.81 (1.55, 2.11)	9.0	-0.10	-14.7	0.02	0.31	75.3	100.0
		All covariates	1.68 (1.44, 1.98)	9.5	-0.17	-24.8	0.04	0.33	42.9	100.0
	Monte Carlo SE (min~max)				0.0022~ 0.0026	0.31~0.37	0.0003~ 0.0015	0.0001~ 0.0001	0.00~ 0.02	0~0
1-6	$OR_{AY} = 5$ ($\beta = 1.6$)	No covariates	6.51 (5.62, 7.54)	7.8	0.26	16.4	0.08	0.30	5.7	100.0
		1 confounder	6.31 (5.45, 7.32)	7.9	0.23	14.5	0.06	0.31	12.8	100.0
		2 confounders	6.12 (5.26, 7.11)	8.1	0.20	12.5	0.05	0.31	26.9	100.0
		3 confounders	5.87 (5.05, 6.83)	8.3	0.16	10.0	0.03	0.31	49.0	100.0
		4 confounders	5.67 (4.86, 6.62)	8.6	0.13	7.9	0.02	0.32	66.3	100.0
		5 confounders	5.42 (4.63, 6.34)	8.8	0.08	5.0	0.01	0.32	85.2	100.0
		6 confounders	5.18 (4.41, 6.09)	9.3	0.04	2.2	0.01	0.33	94.1	100.0
		+ 1 risk factor	5.38 (4.56, 6.34)	9.8	0.07	4.5	0.01	0.34	87.3	100.0
		+ 1 mediator	4.91 (4.17, 5.79)	9.4	-0.02	-1.1	0.01	0.34	94.3	100.0

	+ 1 collider	4.72 (4.01, 5.57)	9.6	-0.06	-3.5	0.01	0.34	90.6	100.0
	All covariates	4.63 (3.90, 5.50)	10.3	-0.08	-4.8	0.01	0.35	86.4	100.0
Monte Carlo SE (min~max)				0.0024~ 0.0028	0.15~0.17	0.0004~ 0.0013	0.0001~ 0.0002	0.01~ 0.02	0~0

OR, odds ratio; CI, confidence interval; MSE, mean square error; *A*, exposure; *Y*, outcome; SE, standard error.
The accuracy of every adjustment strategy was acceptable when OR_{AY} was away from 1, but the accuracy sharply decreased when OR_{AY} was around 1. Only pooling original ORs that fully adjusted for 6 confounders would show precise results for weak or none effect.

Table S4. Performances of statistical adjustment strategies in scenarios with different independent associations of covariates with exposure (OR_{UA}) in target population

Scenario	Description	Adjustment strategy	Pooled $\widehat{OR}$ (95% $\widehat{CI}$)	I^2 (%)	Performance of $\hat{\beta} = \ln(\widehat{OR})$					
					Bias	Relative bias (%)	MSE	Width of CI	Coverage (%)	Power (%)
2-1	OR_{UA} = 0.2	No covariates	1.08 (0.87, 1.34)	5.5	-0.62	-89.0	0.39	0.48	0.1	7.0
	OR_{AY} = 2	1 confounder	1.20 (0.96, 1.49)	5.6	-0.51	-74.0	0.28	0.49	0.9	28.9
	(β = 0.7)	2 confounders	1.32 (1.05, 1.65)	6.0	-0.42	-60.2	0.19	0.49	7.0	60.2
		3 confounders	1.44 (1.15, 1.81)	6.3	-0.33	-47.4	0.12	0.50	25.1	84.0
		4 confounders	1.61 (1.28, 2.02)	6.5	-0.22	-31.7	0.06	0.50	60.3	97.1
		5 confounders	1.82 (1.44, 2.30)	6.6	-0.10	-13.9	0.02	0.51	91.0	99.8
		6 confounders	2.07 (1.64, 2.63)	6.5	0.04	5.3	0.02	0.52	96.4	99.9
		+ 1 risk factor	2.12 (1.67, 2.71)	7.2	0.06	8.7	0.02	0.53	95.0	100.0
		+ 1 mediator	2.40 (1.88, 3.05)	6.7	0.18	26.1	0.05	0.53	73.3	100.0
		+ 1 collider	2.40 (1.88, 3.05)	6.6	0.18	26.2	0.05	0.53	74.8	100.0
		All covariates	2.85 (2.22, 3.67)	7.6	0.35	51.2	0.14	0.54	25.4	100.0
	Monte Carlo SE (min~max)				0.0035~ 0.0040	0.50~0.59	0.0007~ 0.0043	0.0004~ 0.0004	0.00~ 0.02	0~0.02
2-2	OR_{UA} = 0.5	No covariates	1.40 (1.17, 1.68)	7.1	-0.36	-51.7	0.14	0.40	4.1	91.9
	OR_{AY} = 2	1 confounder	1.47 (1.22, 1.77)	7.3	-0.31	-44.1	0.10	0.40	14.2	96.9
	(β = 0.7)	2 confounders	1.53 (1.27, 1.84)	7.3	-0.27	-38.6	0.08	0.41	25.0	99.4
		3 confounders	1.64 (1.36, 1.98)	7.4	-0.20	-28.3	0.05	0.41	54.4	100.0
		4 confounders	1.73 (1.43, 2.09)	7.4	-0.15	-21.0	0.03	0.42	73.2	100.0
		5 confounders	1.90 (1.56, 2.31)	7.5	-0.05	-7.3	0.01	0.42	93.5	100.0
		6 confounders	2.04 (1.67, 2.49)	8.1	0.02	2.8	0.01	0.43	95.9	100.0
		+ 1 risk factor	2.07 (1.69, 2.54)	8.7	0.03	4.8	0.01	0.43	95.8	100.0
		+ 1 mediator	2.19 (1.78, 2.69)	8.4	0.09	12.9	0.02	0.43	88.5	100.0
		+ 1 collider	2.19 (1.79, 2.70)	8.4	0.09	13.4	0.02	0.44	88.6	100.0
		All covariates	2.40 (1.93, 2.97)	9.4	0.18	26.0	0.04	0.45	64.8	100.0
	Monte Carlo SE				0.0029~	0.42~0.50	0.0005~	0.0003~	0.01~	0~0.01

	(min~max)				0.0035		0.0022	0.0003	0.02	
2-3	OR_{UA} = 0.8	No covariates	1.57 (1.33, 1.86)	7.5	-0.24	-34.7	0.07	0.35	22.3	100.0
	OR_{AY} = 2	1 confounder	1.61 (1.36, 1.91)	7.7	-0.22	-31.4	0.05	0.35	29.8	100.0
	(β = 0.7)	2 confounders	1.68 (1.41, 1.99)	7.9	-0.18	-25.4	0.04	0.35	52.3	100.0
		3 confounders	1.73 (1.45, 2.07)	8.1	-0.14	-20.8	0.03	0.36	65.4	100.0
		4 confounders	1.79 (1.49, 2.13)	8.0	-0.11	-16.4	0.02	0.36	76.7	100.0
		5 confounders	1.87 (1.56, 2.24)	8.3	-0.07	-9.8	0.01	0.37	89.5	100.0
		6 confounders	1.96 (1.63, 2.36)	8.4	-0.02	-2.8	0.01	0.37	95.6	100.0
		+ 1 risk factor	2.00 (1.66, 2.41)	9.1	0.00	0.1	0.01	0.38	95.7	100.0
		+ 1 mediator	2.03 (1.69, 2.45)	8.4	0.02	2.3	0.01	0.38	94.8	100.0
		+ 1 collider	2.06 (1.71, 2.48)	8.7	0.03	4.2	0.01	0.38	94.3	100.0
		All covariates	2.18 (1.80, 2.64)	9.4	0.09	12.3	0.02	0.39	87.1	100.0
	Monte Carlo SE				0.0027~	0.39~0.45	0.0004~	0.0002~	0.01~	0~0
	(min~max)				0.0031		0.0013	0.0002	0.02	
2-4	OR_{UA} = 1	No covariates	1.84 (1.58, 2.15)	7.4	-0.08	-11.9	0.01	0.32	83.5	100.0
	OR_{AY} = 2	1 confounder	1.89 (1.62, 2.21)	7.5	-0.06	-8.2	0.01	0.32	89.6	100.0
	(β = 0.7)	2 confounders	1.89 (1.62, 2.22)	7.6	-0.05	-7.8	0.01	0.33	89.9	100.0
		3 confounders	1.93 (1.65, 2.27)	7.8	-0.03	-4.8	0.01	0.33	94.0	100.0
		4 confounders	1.96 (1.67, 2.31)	8.2	-0.02	-2.6	0.01	0.34	95.6	100.0
		5 confounders	2.00 (1.69, 2.36)	8.3	-0.00	-0.1	0.01	0.34	95.9	100.0
		6 confounders	2.02 (1.71, 2.38)	8.4	0.01	1.2	0.01	0.34	95.7	100.0
		+ 1 risk factor	2.05 (1.73, 2.44)	8.5	0.03	3.9	0.01	0.35	94.5	100.0
		+ 1 mediator	2.03 (1.71, 2.41)	8.5	0.01	2.1	0.01	0.35	95.4	100.0
		+ 1 collider	2.03 (1.71, 2.41)	8.9	0.02	2.4	0.01	0.35	95.3	100.0
		All covariates	2.09 (1.74, 2.49)	9.1	0.04	6.0	0.01	0.36	93.1	100.0
	Monte Carlo SE				0.0025~	0.36~0.42	0.0003~	0.0001~	0.01~	0~0
	(min~max)				0.0029		0.0005	0.0002	0.01	
2-5	OR_{UA} = 1.25	No covariates	2.10 (1.81, 2.43)	7.6	0.05	6.9	0.01	0.30	91.5	100.0
	OR_{AY} = 2	1 confounder	2.10 (1.81, 2.43)	7.8	0.05	6.8	0.01	0.30	92.2	100.0
	(β = 0.7)	2 confounders	2.07 (1.79, 2.41)	8.1	0.04	5.3	0.01	0.30	93.1	100.0
		3 confounders	2.06 (1.78, 2.39)	8.3	0.03	4.5	0.01	0.31	94.5	100.0

		4 confounders	2.03 (1.75, 2.37)	8.3	0.02	2.4	0.01	0.31	95.7	100.0
		5 confounders	2.01 (1.72, 2.34)	8.9	0.00	0.6	0.01	0.31	96.0	100.0
		6 confounders	1.99 (1.71, 2.33)	9.2	-0.00	-0.6	0.01	0.32	96.3	100.0
		+ 1 risk factor	2.03 (1.73, 2.37)	9.5	0.01	1.9	0.01	0.32	95.8	100.0
		+ 1 mediator	1.95 (1.67, 2.82)	9.4	-0.03	-3.6	0.01	0.32	95.4	100.0
		+ 1 collider	1.91 (1.63, 2.24)	9.4	-0.05	-6.7	0.01	0.33	91.3	100.0
		All covariates	1.90 (1.61, 2.24)	9.9	-0.05	-7.5	0.01	0.34	89.9	100.0
	Monte Carlo SE (min~max)				0.0023~ 0.0027	0.34~0.38	0.0003~ 0.0004	0.0001~ 0.0001	0.01~ 0.01	0~0
Ref	OR_{UA} = 2	No covariates	2.82 (2.46, 3.22)	7.5	0.34	49.4	0.12	0.27	0.1	100.0
	OR_{AY} = 2	1 confounder	2.68 (2.34, 3.07)	7.8	0.29	42.4	0.09	0.27	1.7	100.0
	(β = 0.7)	2 confounders	2.53 (2.21, 2.91)	8.1	0.24	34.1	0.06	0.28	7.4	100.0
		3 confounders	2.41 (2.09, 2.78)	8.1	0.19	27.0	0.04	0.28	25.5	100.0
		4 confounders	2.28 (1.98, 2.63)	8.3	0.13	19.0	0.02	0.29	57.3	100.0
		5 confounders	2.16 (1.87, 2.50)	8.5	0.08	11.4	0.01	0.29	81.5	100.0
		6 confounders	2.01 (1.74, 2.34)	8.8	0.01	1.0	0.01	0.30	94.0	100.0
		+ 1 risk factor	2.05 (1.76, 2.38)	9.3	0.02	3.4	0.01	0.30	93.5	100.0
		+ 1 mediator	1.86 (1.59, 2.16)	8.9	-0.07	-10.8	0.01	0.31	83.7	100.0
		+ 1 collider	1.81 (1.55, 2.11)	9.0	-0.10	-14.7	0.02	0.31	75.3	100.0
		All covariates	1.68 (1.44, 1.98)	9.5	-0.17	-24.8	0.04	0.33	42.9	100.0
	Monte Carlo SE (min~max)				0.0022~ 0.0026	0.31~0.37	0.0003~ 0.0015	0.0001~ 0.0001	0.00~ 0.02	0~0
2-6	OR_{UA} = 5	No covariates	3.81 (3.35, 4.33)	8.2	0.64	93.0	0.42	0.27	0	100.0
	OR_{AY} = 2	1 confounder	3.54 (3.10, 4.03)	8.3	0.57	82.2	0.33	0.27	0	100.0
	(β = 0.7)	2 confounders	3.18 (2.78, 3.64)	8.4	0.46	67.1	0.22	0.28	0	100.0
		3 confounders	2.85 (2.47, 3.27)	8.4	0.35	50.9	0.13	0.29	0.3	100.0
		4 confounders	2.55 (2.21, 2.95)	8.6	0.24	35.2	0.07	0.30	9.9	100.0
		5 confounders	2.30 (1.98, 2.67)	9.0	0.14	19.9	0.02	0.31	57.8	100.0
		6 confounders	2.04 (1.75, 2.39)	8.7	0.02	3.1	0.01	0.32	94.8	100.0
		+ 1 risk factor	2.12 (1.81, 2.48)	8.9	0.06	8.2	0.01	0.33	91.2	100.0
		+ 1 mediator	1.64 (1.39, 1.94)	9.1	-0.20	-28.3	0.05	0.34	38.1	100.0

	+ 1 collider	1.61 (1.36, 1.90)	8.7	-0.22	-31.7	0.06	0.34	29.9	100.0
	All covariates	1.31 (1.09, 1.57)	9.6	-0.43	-61.5	0.19	0.37	0.6	79.6
Monte Carlo SE (min~max)				0.0021~ 0.0029	0.30~0.42	0.0003~ 0.0027	0.0001~ 0.0001	0~0.02	0~0

OR, odds ratio; CI, confidence interval; MSE, mean square error; *A*, exposure; *Y*, outcome; ***U***, covariate; SE, standard error.
The effect estimation of every adjustment strategy was rarely affected by OR_{UA}, unless OR_{UA} was strong.

Table S5. Performances of statistical adjustment strategies in scenarios with different independent associations of covariates with outcome (OR_{UY}) in target population

Scenario	Description	Adjustment strategy	Pooled $\widehat{OR}$ (95% $\widehat{CI}$)	I^2 (%)	Performance of $\hat{\beta} = \ln(\widehat{OR})$					
					Bias	Relative bias (%)	MSE	Width of CI	Coverage (%)	Power (%)
3-1	OR_{UY} = 0.2	No covariates	1.01 (0.90, 1.14)	5.3	-0.68	-98.5	0.47	0.26	0	2.6
	OR_{AY} = 2	1 confounder	1.14 (1.01, 1.30)	5.5	-0.56	-80.6	0.32	0.27	0	51.1
	(β = 0.7)	2 confounders	1.32 (1.15, 1.51)	6.0	-0.42	-60.2	0.18	0.28	0	96.6
		3 confounders	1.45 (1.25, 1.67)	5.7	-0.32	-46.6	0.11	0.29	0.7	99.9
		4 confounders	1.59 (1.37, 1.84)	6.4	-0.23	-33.3	0.06	0.30	15.7	100.0
		5 confounders	1.78 (1.53, 2.08)	6.3	-0.12	-16.7	0.02	0.32	68.4	100.0
		6 confounders	1.99 (1.70, 2.34)	6.6	-0.00	-0.5	0.01	0.33	95.6	100.0
		+ 1 risk factor	1.98 (1.67, 2.36)	6.9	-0.01	-1.3	0.01	0.35	95.9	100.0
		+ 1 mediator	2.50 (2.10, 2.98)	7.1	0.33	32.1	0.06	0.36	29.9	100.0
		+ 1 collider	2.27 (1.92, 2.68)	6.5	0.12	18.0	0.02	0.35	71.8	100.0
		All covariates	2.88 (2.38, 3.50)	7.2	0.37	52.7	0.14	0.40	4.7	100.0
	Monte Carlo SE (min~max)				0.0020~ 0.0031	0.28~0.45	0.0003~ 0.0027	0.0000~ 0.0002	0~0.01	0~0.02
3-2	OR_{UY} = 0.5	No covariates	1.33 (1.17, 1.51)	7.4	-0.41	-59.3	0.17	0.25	0	99.3
	OR_{AY} = 2	1 confounder	1.40 (1.23, 1.60)	7.4	-0.36	-51.3	0.13	0.26	0	99.9
	(β = 0.7)	2 confounders	1.50 (1.32, 1.72)	7.5	-0.28	-41.0	0.09	0.27	1.2	100.0
		3 confounders	1.61 (1.41, 1.84)	7.4	-0.22	-31.3	0.05	0.27	12.8	100.0
		4 confounders	1.79 (1.55, 2.06)	7.6	-0.11	-16.2	0.02	0.28	65.2	100.0
		5 confounders	1.95 (1.69, 2.25)	7.9	-0.02	-3.5	0.01	0.29	94.7	100.0
		6 confounders	2.04 (1.77, 2.36)	8.1	0.02	3.1	0.01	0.30	94.8	100.0
		+ 1 risk factor	2.06 (1.77, 2.38)	8.2	0.03	3.9	0.01	0.30	93.3	100.0
		+ 1 mediator	2.23 (1.92, 2.58)	8.2	0.11	15.6	0.02	0.30	70.7	100.0
		+ 1 collider	2.17 (1.87, 2.51)	8.1	0.08	11.6	0.01	0.30	82.0	100.0
		All covariates	2.37 (2.03, 2.77)	8.5	0.17	24.7	0.04	0.31	43.4	100.0
	Monte Carlo SE				0.0021~	0.30~0.36	0.0003~	0.0000~	0~0.02	0~0.00

	(min~max)				0.0025		0.0017	0.0001		
3-3	OR_{UY} = 0.8	No covariates	1.72 (1.51, 1.95)	7.4	-0.15	-22.1	0.03	0.26	34.9	100.0
	OR_{AY} = 2	1 confounder	1.78 (1.56, 2.02)	7.6	-0.12	-17.1	0.02	0.26	56.2	100.0
	(β = 0.7)	2 confounders	1.83 (1.61, 2.09)	8.0	-0.09	-12.7	0.01	0.26	74.7	100.0
		3 confounders	1.88 (1.64, 2.14)	8.1	-0.06	-9.3	0.01	0.27	84.3	100.0
		4 confounders	1.95 (1.70, 2.24)	8.3	-0.02	-3.5	0.01	0.27	93.4	100.0
		5 confounders	2.00 (1.74, 2.30)	8.4	-0.00	-0.1	0.01	0.28	94.8	100.0
		6 confounders	2.07 (1.79, 2.38)	8.6	0.03	4.7	0.01	0.28	93.2	100.0
		+ 1 risk factor	2.09 (1.81, 2.42)	9.0	0.05	6.6	0.01	0.29	91.0	100.0
		+ 1 mediator	2.13 (1.84, 2.46)	8.5	0.06	9.0	0.01	0.29	87.6	100.0
		+ 1 collider	2.13 (1.84, 2.47)	8.7	0.06	9.4	0.01	0.29	86.5	100.0
		All covariates	2.23 (1.92, 2.59)	8.9	0.11	15.6	0.02	0.30	69.4	100.0
	Monte Carlo SE				0.0020~	0.29~0.35	0.0003~	0.0000~	0.01~	0~0
	(min~max)				0.0024		0.0006	0.0001	0.02	
3-4	OR_{UY} = 1	No covariates	1.98 (1.75, 2.24)	7.7	-0.01	-1.3	0.00	0.26	95.4	100.0
	OR_{AY} = 2	1 confounder	2.00 (1.76, 2.27)	8.0	-0.00	-0.3	0.00	0.26	95.2	100.0
	(β = 0.7)	2 confounders	2.00 (1.75, 2.27)	8.1	-0.00	-0.3	0.00	0.26	95.6	100.0
		3 confounders	2.01 (1.76, 2.29)	8.4	0.00	0.6	0.00	0.27	95.8	100.0
		4 confounders	2.02 (1.76, 2.31)	8.5	0.01	1.1	0.00	0.27	95.7	100.0
		5 confounders	2.02 (1.76, 2.31)	8.8	0.01	1.5	0.00	0.28	95.8	100.0
		6 confounders	2.03 (1.77, 2.33)	9.0	0.01	2.1	0.01	0.28	94.9	100.0
		+ 1 risk factor	2.04 (1.77, 2.34)	9.1	0.02	2.5	0.01	0.28	94.6	100.0
		+ 1 mediator	2.01 (1.75, 2.31)	9.2	0.01	0.8	0.01	0.29	94.7	100.0
		+ 1 collider	2.07 (1.80, 2.38)	9.1	0.03	4.7	0.01	0.29	92.8	100.0
		All covariates	2.05 (1.78, 2.37)	9.6	0.03	3.9	0.01	0.29	93.2	100.0
	Monte Carlo SE				0.0020~	0.29~0.33	0.0002~	0.0000~	0.01~	0~0
	(min~max)				0.0023		0.0003	0.0001	0.01	
3-5	OR_{UY} = 1.25	No covariates	2.32 (2.03, 2.65)	8.4	0.15	21.4	0.03	0.26	41.0	100.0
	OR_{AY} = 2	1 confounder	2.28 (2.00, 2.61)	8.6	0.13	19.2	0.02	0.26	50.1	100.0
	(β = 0.7)	2 confounders	2.25 (1.96, 2.58)	8.8	0.12	17.0	0.02	0.27	59.3	100.0
		3 confounders	2.18 (1.90, 2.50)	8.9	0.09	12.5	0.01	0.27	76.9	100.0

		4 confounders	2.14 (1.86, 2.46)	9.2	0.07	9.5	0.01	0.27	84.3	100.0
		5 confounders	2.10 (1.82, 2.42)	9.4	0.05	7.2	0.01	0.28	89.0	100.0
		6 confounders	2.05 (1.77, 2.37)	9.7	0.03	3.7	0.01	0.28	93.3	100.0
		+ 1 risk factor	2.07 (1.79, 2.40)	9.9	0.04	5.3	0.01	0.28	91.6	100.0
		+ 1 mediator	1.98 (1.71, 2.30)	9.8	-0.01	-1.4	0.01	0.29	94.5	100.0
		+ 1 collider	2.00 (1.72, 2.32)	10.1	-0.00	-0.1	0.01	0.29	94.6	100.0
		All covariates	1.95 (1.68, 2.27)	10.4	-0.02	-3.5	0.01	0.29	93.2	100.0
	Monte Carlo SE (min~max)				0.0021~ 0.0025	0.31~0.36	0.0003~ 0.0007	0.0000~ 0.0001	0.01~ 0.02	0~0
Ref	OR_{UY} = 2	No covariates	2.82 (2.46, 3.22)	7.5	0.34	49.4	0.12	0.27	0.1	100.0
	OR_{AY} = 2	1 confounder	2.68 (2.34, 3.07)	7.8	0.29	42.4	0.09	0.27	1.7	100.0
	(β = 0.7)	2 confounders	2.53 (2.21, 2.91)	8.1	0.24	34.1	0.06	0.28	7.4	100.0
		3 confounders	2.41 (2.09, 2.78)	8.1	0.19	27.0	0.04	0.28	25.5	100.0
		4 confounders	2.28 (1.98, 2.63)	8.3	0.13	19.0	0.02	0.29	57.3	100.0
		5 confounders	2.16 (1.87, 2.50)	8.5	0.08	11.4	0.01	0.29	81.5	100.0
		6 confounders	2.01 (1.74, 2.34)	8.8	0.01	1.0	0.01	0.30	94.0	100.0
		+ 1 risk factor	2.05 (1.76, 2.38)	9.3	0.02	3.4	0.01	0.30	93.5	100.0
		+ 1 mediator	1.86 (1.59, 2.16)	8.9	-0.07	-10.8	0.01	0.31	83.7	100.0
		+ 1 collider	1.81 (1.55, 2.11)	9.0	-0.10	-14.7	0.02	0.31	75.3	100.0
		All covariates	1.68 (1.44, 1.98)	9.5	-0.17	-24.8	0.04	0.33	42.9	100.0
	Monte Carlo SE (min~max)				0.0022~ 0.0026	0.31~0.37	0.0003~ 0.0015	0.0001~ 0.0001	0.00~ 0.02	0~0
3-6	OR_{UY} = 5	No covariates	3.41 (2.97, 3.91)	7.7	0.53	76.8	0.29	0.09	0	100.0
	OR_{AY} = 2	1 confounder	3.18 (2.76, 3.67)	7.8	0.46	67.1	0.22	0.29	0	100.0
	(β = 0.7)	2 confounders	2.92 (2.51, 3.39)	7.9	0.38	54.6	0.15	0.30	0.1	100.0
		3 confounders	2.74 (2.35, 3.20)	7.8	0.32	45.6	0.11	0.31	2.2	100.0
		4 confounders	2.55 (2.17, 3.00)	8.0	0.24	35.2	0.07	0.33	15.3	100.0
		5 confounders	2.35 (1.99, 2.77)	8.0	0.16	23.3	0.03	0.33	54.1	100.0
		6 confounders	2.11 (1.77, 2.51)	8.0	0.05	7.6	0.01	0.35	91.3	100.0
		+ 1 risk factor	2.10 (1.75, 2.52)	8.7	0.05	7.2	0.01	0.36	92.3	100.0
		+ 1 mediator	1.84 (1.54, 2.21)	8.8	-0.08	-11.9	0.02	0.36	86.2	100.0

	+ 1 collider	1.73 (1.44, 2.09)	8.6	-0.14	-20.7	0.03	0.38	67.4	100.0
	All covariates	1.48 (1.21, 1.82)	9.6	-0.30	-43.5	0.10	0.42	18.2	95.3
Monte Carlo SE (min~max)				0.0022~ 0.0033	0.32~0.48	0.0005~ 0.0024	0.0001~ 0.0002	0~0.02	0~0

OR, odds ratio; CI, confidence interval; MSE, mean square error; *A*, exposure; *Y*, outcome; ***U***, covariate; SE standard error.
The effect estimation of every adjustment strategy was rarely affected by OR_{UY}, unless OR_{UY} was strong.

Table S6. Performances of statistical adjustment strategies in scenarios with different correlation coefficients among covariates (r_{UU}) in target population

Scenario	Description	Adjustment strategy	Pooled $\widehat{OR}$ (95% $\widehat{CI}$)	I^2 (%)	Performance of $\hat{\beta} = \ln(\widehat{OR})$					
					Bias	Relative bias (%)	MSE	Width of CI	Coverage (%)	Power (%)
Ref	$r_{UU} = 0$	No covariates	2.82 (2.46, 3.22)	7.5	0.34	49.4	0.12	0.27	0.1	100.0
	$OR_{AY} = 2$	1 confounder	2.68 (2.34, 3.07)	7.8	0.29	42.4	0.09	0.27	1.7	100.0
	($\beta = 0.7$)	2 confounders	2.53 (2.21, 2.91)	8.1	0.24	34.1	0.06	0.28	7.4	100.0
		3 confounders	2.41 (2.09, 2.78)	8.1	0.19	27.0	0.04	0.28	25.5	100.0
		4 confounders	2.28 (1.98, 2.63)	8.3	0.13	19.0	0.02	0.29	57.3	100.0
		5 confounders	2.16 (1.87, 2.50)	8.5	0.08	11.4	0.01	0.29	81.5	100.0
		6 confounders	2.01 (1.74, 2.34)	8.8	0.01	1.0	0.01	0.30	94.0	100.0
		+ 1 risk factor	2.05 (1.76, 2.38)	9.3	0.02	3.4	0.01	0.30	93.5	100.0
		+ 1 mediator	1.86 (1.59, 2.16)	8.9	-0.07	-10.8	0.01	0.31	83.7	100.0
		+ 1 collider	1.81 (1.55, 2.11)	9.0	-0.10	-14.7	0.02	0.31	75.3	100.0
		All covariates	1.68 (1.44, 1.98)	9.5	-0.17	-24.8	0.04	0.33	42.9	100.0
	Monte Carlo SE (min~max)				0.0022~ 0.0026	0.31~0.37	0.0003~ 0.0015	0.0001~ 0.0001	0.00~ 0.02	0~0
4-1	$r_{UU} = 0.2$	No covariates	3.52 (3.08, 4.02)	8.0	0.56	81.5	0.32	0.27	0	100.0
	$OR_{AY} = 2$	1 confounder	2.98 (2.61, 3.42)	8.2	0.40	57.7	0.16	0.28	0.1	100.0
	($\beta = 0.7$)	2 confounders	2.67 (2.32, 3.08)	8.3	0.29	41.9	0.09	0.29	2.1	100.0
		3 confounders	2.43 (2.10, 2.80)	8.4	0.19	28.0	0.04	0.30	28.0	100.0
		4 confounders	2.24 (1.93, 2.60)	8.3	0.11	16.5	0.02	0.31	68.6	100.0
		5 confounders	2.11 (1.81, 2.46)	8.8	0.05	7.8	0.01	0.31	91.1	100.0
		6 confounders	2.01 (1.72, 2.35)	9.2	0.01	1.0	0.01	0.32	95.5	100.0
		+ 1 risk factor	2.07 (1.77, 2.42)	9.2	0.03	5.0	0.01	0.32	93.3	100.0
		+ 1 mediator	1.87 (1.60, 2.20)	9.4	-0.06	-9.3	0.01	0.33	89.1	100.0
		+ 1 collider	1.83 (1.56, 2.15)	9.3	-0.09	-13.0	0.01	0.33	81.0	100.0
		All covariates	1.74 (1.48, 2.06)	9.6	-0.14	-19.8	0.03	0.34	66.0	100.0
	Monte Carlo SE				0.0021~	0.31~0.39	0.0003~	0.0001~	0~0.01	0~0

	(min~max)				0.0027		0.0024	0.0001		
4-2	$r_{UU} = 0.5$	No covariates	4.68 (4.08, 5.38)	8.7	0.85	122.8	0.73	0.28	0	100.0
	$OR_{AY} = 2$	1 confounder	3.04 (2.62, 3.53)	9.2	0.42	60.6	0.18	0.30	0.2	100.0
	($\beta = 0.7$)	2 confounders	2.57 (2.20, 2.99)	8.9	0.25	36.0	0.07	0.32	12.9	100.0
		3 confounders	2.33 (1.99, 2.73)	8.6	0.15	22.2	0.03	0.32	53.2	100.0
		4 confounders	2.18 (1.85, 2.56)	8.8	0.09	12.4	0.01	0.33	81.9	100.0
		5 confounders	2.09 (1.77, 2.47)	9.2	0.04	6.4	0.01	0.34	91.7	100.0
		6 confounders	2.03 (1.72, 2.41)	9.4	0.02	2.4	0.01	0.34	95.8	100.0
		+ 1 risk factor	2.09 (1.76, 2.48)	9.5	0.04	6.2	0.01	0.35	92.4	100.0
		+ 1 mediator	1.91 (1.61, 2.27)	9.8	-0.05	-6.7	0.01	0.35	92.3	100.0
		+ 1 collider	1.80 (1.51, 2.14)	9.7	-0.10	-15.1	0.02	0.35	78.2	100.0
		All covariates	1.73 (1.44, 2.07)	10.0	-0.15	-21.3	0.03	0.37	64.7	100.0
	Monte Carlo SE (min~max)				0.0022~ 0.0029	0.32~0.42	0.0003~ 0.0038	0.0001~ 0.0002	0~0.02	0~0
4-3	$r_{UU} = 0.8$	No covariates	5.94 (5.17, 6.83)	8.0	1.09	157.1	1.19	0.29	0	100.0
	$OR_{AY} = 2$	1 confounder	2.59 (2.21, 3.04)	8.9	0.26	37.2	0.07	0.33	12.5	100.0
	($\beta = 0.7$)	2 confounders	2.21 (1.88, 2.60)	8.3	0.10	14.4	0.02	0.34	80.7	100.0
		3 confounders	2.07 (1.75, 2.43)	8.1	0.03	4.7	0.01	0.35	94.6	100.0
		4 confounders	2.03 (1.72, 2.40)	8.2	0.01	2.2	0.01	0.35	95.7	100.0
		5 confounders	2.02 (1.70, 2.39)	8.3	0.01	1.1	0.01	0.36	95.7	100.0
		6 confounders	2.00 (1.69, 2.38)	8.5	0.00	0.19	0.01	0.36	95.5	100.0
		+ 1 risk factor	2.02 (1.70, 2.41)	8.8	0.01	1.8	0.01	0.37	94.9	100.0
		+ 1 mediator	1.85 (1.55, 2.20)	8.1	-0.08	-11.6	0.01	0.37	87.4	100.0
		+ 1 collider	1.78 (1.49, 2.13)	8.5	-0.12	-16.8	0.02	0.37	79.3	100.0
		All covariates	1.65 (1.37, 1.98)	8.3	-0.19	-28.1	0.05	0.39	50.2	100.0
	Monte Carlo SE (min~max)				0.0022~ 0.0030	0.32~0.43	0.0004~ 0.0049	0.0001~ 0.0002	0~0.02	0~0

OR, odds ratio; CI, confidence interval; MSE, mean square error; *A*, exposure; *Y*, outcome; ***U***, covariate; SE, standard error.

If the confounders were correlated ($r_{UU} \neq 0$), less than 6 of them would be sufficient to control confounding bias. Except for unadjustment strategy that greatly overestimated the true effect when r_{UU} was strong, collinearity hardly influence the effect estimations under other adjustment strategies.

Table S7. Performances of statistical adjustment strategies in scenarios with different number of cases (*m*) in original case-control studies

Scenario	Description	Adjustment strategy	Pooled $\widehat{OR}$ (95% $\widehat{CI}$)	I^2 (%)	Performance of $\hat{\beta} = \ln(\widehat{OR})$					
					Bias	Relative bias (%)	MSE	Width of CI	Coverage (%)	Power (%)
5-1	$m = 20$	No covariates	2.77 (2.07, 3.69)	6.7	0.32	46.8	0.13	0.62	46.6	100.0
	$OR_{AY} = 2$	1 confounder	2.69 (1.99, 3.62)	7.1	0.29	42.5	0.11	0.64	58.2	100.0
	($\beta = 0.7$)	2 confounders	2.57 (1.87, 3.53)	7.4	0.25	36.4	0.09	0.67	70.6	100.0
		3 confounders	2.50 (1.79, 3.48)	7.9	0.22	31.9	0.08	0.71	79.6	100.0
		4 confounders	2.40 (1.69, 3.41)	8.3	0.18	26.4	0.07	0.74	85.7	99.8
		5 confounders	2.32 (1.59, 3.38)	9.0	0.15	21.3	0.06	0.78	89.7	99.2
		6 confounders	2.19 (1.46, 3.28)	9.0	0.09	13.0	0.05	0.83	94.7	96.0
		+ 1 risk factor	2.26 (1.46, 3.51)	9.9	0.12	17.9	0.07	0.89	92.1	95.3
		+ 1 mediator	2.04 (1.33, 3.12)	9.6	0.02	2.7	0.05	0.90	96.4	88.3
		+ 1 collider	1.97 (1.26, 3.07)	9.4	-0.02	-2.2	0.05	0.90	95.2	84.3
		All covariates	1.86 (1.11, 3.14)	9.2	-0.07	-10.2	0.08	1.05	94.6	62.9
	Monte Carlo SE (min~max)				0.0046~ 0.0084	0.67~1.22	0.0022~ 0.0033	0.0004~ 0.0019	0.01~ 0.02	0~0.2
Ref	$m = 100$	No covariates	2.82 (2.46, 3.22)	7.5	0.34	49.4	0.12	0.27	0.1	100.0
	$OR_{AY} = 2$	1 confounder	2.68 (2.34, 3.07)	7.8	0.29	42.4	0.09	0.27	1.7	100.0
	($\beta = 0.7$)	2 confounders	2.53 (2.21, 2.91)	8.1	0.24	34.1	0.06	0.28	7.4	100.0
		3 confounders	2.41 (2.09, 2.78)	8.1	0.19	27.0	0.04	0.28	25.5	100.0
		4 confounders	2.28 (1.98, 2.63)	8.3	0.13	19.0	0.02	0.29	57.3	100.0
		5 confounders	2.16 (1.87, 2.50)	8.5	0.08	11.4	0.01	0.29	81.5	100.0
		6 confounders	2.01 (1.74, 2.34)	8.8	0.01	1.0	0.01	0.30	94.0	100.0
		+ 1 risk factor	2.05 (1.76, 2.38)	9.3	0.02	3.4	0.01	0.30	93.5	100.0
		+ 1 mediator	1.86 (1.59, 2.16)	8.9	-0.07	-10.8	0.01	0.31	83.7	100.0
		+ 1 collider	1.81 (1.55, 2.11)	9.0	-0.10	-14.7	0.02	0.31	75.3	100.0
		All covariates	1.68 (1.44, 1.98)	9.5	-0.17	-24.8	0.04	0.33	42.9	100.0
	Monte Carlo SE (min~max)				0.0022~ 0.0026	0.31~0.37	0.0003~ 0.0015	0.0001~ 0.0001	0.00~ 0.02	0~0

5-2	m = 500	No covariates	2.82 (2.67, 2.98)	5.8	0.34	49.6	0.12	0.12	0	100.0
	OR_{AY} = 2	1 confounder	2.68 (2.53, 2.83)	5.7	0.29	42.0	0.09	0.12	0	100.0
	(β = 0.7)	2 confounders	2.52 (2.38, 2.67)	5.8	0.23	33.2	0.05	0.12	0	100.0
		3 confounders	2.39 (2.25, 2.53)	5.7	0.18	25.6	0.03	0.12	0	100.0
		4 confounders	2.25 (2.12, 2.39)	5.8	0.12	17.1	0.01	0.13	3.9	100.0
		5 confounders	2.13 (2.01, 2.26)	5.9	0.06	9.1	0.00	0.13	51.5	100.0
		6 confounders	1.98 (1.86, 2.10)	5.8	-0.01	-1.6	0.00	0.13	94.3	100.0
		+ 1 risk factor	2.00 (1.89, 2.13)	5.9	0.00	0.3	0.00	0.13	97.0	100.0
		+ 1 mediator	1.82 (1.71, 1.93)	5.9	-0.10	-13.7	0.01	0.13	17.0	100.0
		+ 1 collider	1.77 (1.67, 1.88)	5.8	-0.12	-17.5	0.02	0.13	3.5	100.0
		All covariates	1.65 (1.54, 1.75)	5.8	-0.20	-28.2	0.04	0.14	0	100.0
	Monte Carlo SE (min~max)				0.0010~ 0.0013	0.13~0.15	0.0004~ 0.0006	0.0001~ 0.0002	0~0.02	0~0

OR, odds ratio; CI, confidence interval; MSE, mean square error; *A*, exposure; *Y*, outcome; SE, standard error.

Sample sizes of case-control studies had little impact on the precision of pooled ORs. Only when the number of cases was small, sample size might not have enough power to adjust for too many covariates, and the problem of overfitting led to less stability in effect estimations.

Table S8. Performances of statistical adjustment strategies in scenarios with different matching approach and matching ratio in original case-control studies

Scenario	Description	Adjustment strategy	Pooled $\widehat{OR}$ (95% $\widehat{CI}$)	I^2 (%)	Performance of $\hat{\beta} = \ln(\widehat{OR})$					
					Bias	Relative bias (%)	MSE	Width of CI	Coverage (%)	Power (%)
Ref	Frequency matching $OR_{AY} = 2$ ($\beta = 0.7$)	No covariates	2.82 (2.46, 3.22)	7.5	0.34	49.4	0.12	0.27	0.1	100.0
		1 confounder	2.68 (2.34, 3.07)	7.8	0.29	42.4	0.09	0.27	1.7	100.0
		2 confounders	2.53 (2.21, 2.91)	8.1	0.24	34.1	0.06	0.28	7.4	100.0
		3 confounders	2.41 (2.09, 2.78)	8.1	0.19	27.0	0.04	0.28	25.5	100.0
		4 confounders	2.28 (1.98, 2.63)	8.3	0.13	19.0	0.02	0.29	57.3	100.0
		5 confounders	2.16 (1.87, 2.50)	8.5	0.08	11.4	0.01	0.29	81.5	100.0
		6 confounders	2.01 (1.74, 2.34)	8.8	0.01	1.0	0.01	0.30	94.0	100.0
		+ 1 risk factor	2.05 (1.76, 2.38)	9.3	0.02	3.4	0.01	0.30	93.5	100.0
		+ 1 mediator	1.86 (1.59, 2.16)	8.9	-0.07	-10.8	0.01	0.31	83.7	100.0
		+ 1 collider	1.81 (1.55, 2.11)	9.0	-0.10	-14.7	0.02	0.31	75.3	100.0
		All covariates	1.68 (1.44, 1.98)	9.5	-0.17	-24.8	0.04	0.33	42.9	100.0
	Monte Carlo SE (min~max)				0.0022~ 0.0026	0.31~0.37	0.0003~ 0.0015	0.0001~ 0.0001	0.00~ 0.02	0~0
6-1	1:1 matching $OR_{AY} = 2$ ($\beta = 0.7$)	No covariates	2.61 (2.27, 3.00)	6.9	0.27	38.3	0.08	0.28	4.1	100.0
		1 confounder	2.49 (2.15, 2.87)	7.3	0.22	31.5	0.05	0.29	16.3	100.0
		2 confounders	2.35 (2.02, 2.72)	7.5	0.16	23.0	0.03	0.30	45.2	100.0
		3 confounders	2.23 (1.92, 2.60)	7.8	0.11	15.9	0.02	0.31	72.4	100.0
		4 confounders	2.11 (1.80, 2.48)	8.2	0.06	8.0	0.01	0.32	90.6	100.0
		5 confounders	2.01 (1.71, 2.37)	8.7	0.00	0.6	0.01	0.33	94.7	100.0
		+ 1 risk factor	2.05 (1.73, 2.43)	9.3	0.03	3.8	0.01	0.34	94.1	100.0
		+ 1 mediator	1.85 (1.56, 2.20)	9.1	-0.08	-11.0	0.01	0.34	85.2	100.0
		+ 1 collider	1.81 (1.53, 2.15)	9.3	-0.10	-14.5	0.02	0.34	78.8	100.0
		All covariates	1.69 (1.41, 2.04)	10.3	-0.17	-24.1	0.04	0.37	57.0	100.0
	Monte Carlo SE (min~max)				0.0023~ 0.0030	0.32~0.43	0.0003~ 0.0012	0.0002~ 0.0003	0.01~ 0.02	0~0

6-2	1:2	No covariates	2.64 (2.35, 2.97)	7.5	0.28	40.2	0.08	0.23	0.2	100.0
	matching	1 confounder	2.51 (2.22, 2.82)	7.8	0.23	32.5	0.05	0.24	4.0	100.0
	$OR_{AY} = 2$	2 confounders	2.36 (2.09, 2.67)	8.0	0.17	23.9	0.03	0.25	24.0	100.0
	($\beta = 0.7$)	3 confounders	2.24 (1.98, 2.54)	8.2	0.11	16.4	0.02	0.25	58.7	100.0
		4 confounders	2.11 (1.86, 2.40)	8.3	0.06	8.0	0.01	0.26	86.8	100.0
		5 confounders	2.00 (1.75, 2.28)	8.5	-0.00	-0.2	0.00	0.26	94.9	100.0
		+ 1 risk factor	2.03 (1.77, 2.32)	9.1	0.01	2.1	0.01	0.27	94.4	100.0
		+ 1 mediator	1.84 (1.60, 2.11)	8.9	-0.08	-12.3	0.01	0.27	75.9	100.0
		+ 1 collider	1.79 (1.56, 2.06)	8.6	-0.11	-15.7	0.02	0.27	64.8	100.0
		All covariates	1.67 (1.44, 1.93)	9.5	-0.18	-26.3	0.04	0.29	29.9	100.0
	Monte Carlo SE (min~max)				0.0019~ 0.0024	0.27~0.34	0.0002~ 0.0011	0.0001~ 0.0001	0.00~ 0.02	0~0
6-3	1:4	No covariates	2.66 (2.41, 2.92)	8.2	0.28	40.9	0.08	0.21	0	100.0
	matching	1 confounder	2.51 (2.28, 2.77)	8.5	0.23	32.9	0.05	0.21	0.7	100.0
	$OR_{AY} = 2$	2 confounders	2.37 (2.15, 2.61)	8.4	0.17	24.4	0.03	0.21	10.5	100.0
	($\beta = 0.7$)	3 confounders	2.24 (2.03, 2.48)	8.4	0.12	16.7	0.02	0.22	44.6	100.0
		4 confounders	2.12 (1.91, 2.34)	8.5	0.06	8.2	0.01	0.22	86.0	100.0
		5 confounders	2.00 (1.80, 2.22)	8.7	-0.00	-0.3	0.00	0.23	96.5	100.0
		+ 1 risk factor	2.02 (1.81, 2.25)	8.7	0.01	1.3	0.00	0.23	95.9	100.0
		+ 1 mediator	1.83 (1.64, 2.04)	9.0	-0.09	-12.7	0.01	0.23	70.0	100.0
		+ 1 collider	1.79 (1.60, 1.99)	8.9	-0.11	-16.4	0.02	0.23	53.7	100.0
		All covariates	1.65 (1.47, 1.85)	9.3	-0.19	-27.7	0.04	0.24	11.0	100.0
	Monte Carlo SE (min~max)				0.0015~ 0.0018	0.22~0.26	0.0001~ 0.0009	0.0000~ 0.0001	0~0.02	0~0

OR, odds ratio; CI, confidence interval; MSE, mean square error; *A*, exposure; *Y*, outcome; SE, standard error.
Matching approaches of case-control studies had little impact on the precision of pooled ORs. For individual matched case-control studies, crude ORs have already controlled the matching variable (L_6), and thus the accuracy was better than crude ORs of frequency matched studies.

Table S9. Performances of statistical adjustment strategies in scenarios with different number of case-control studies (N) in meta-analyses

Scenario	Description	Adjustment strategy	Pooled $\widehat{OR}$ (95% $\widehat{CI}$)	I^2 (%)	Performance of $\hat{\beta} = \ln(\widehat{OR})$					
					Bias	Relative bias (%)	MSE	Width of CI	Coverage (%)	Power (%)
7-1	$N = 5$	No covariates	2.83 (2.15, 3.71)	13.6	0.35	49.8	0.14	0.54	28.7	100.0
	$OR_{AY} = 2$	1 confounder	2.69 (2.04, 3.55)	13.7	0.30	42.9	0.11	0.55	43.0	100.0
	($\beta = 0.7$)	2 confounders	2.54 (1.91, 3.38)	14.1	0.24	34.6	0.08	0.56	60.1	100.0
		3 confounders	2.42 (1.81, 3.23)	14.5	0.19	27.3	0.06	0.57	74.3	100.0
		4 confounders	2.28 (1.69, 3.08)	14.6	0.13	19.2	0.04	0.58	84.4	100.0
		5 confounders	2.17 (1.59, 2.94)	14.9	0.08	11.5	0.03	0.59	90.2	99.9
		6 confounders	2.01 (1.47, 2.75)	15.1	0.01	0.7	0.03	0.60	93.9	99.5
		+ 1 risk factor	2.04 (1.49, 2.81)	15.8	0.02	3.0	0.03	0.61	93.9	99.3
		+ 1 mediator	1.85 (1.34, 2.55)	15.4	-0.08	-11.1	0.03	0.61	90.5	97.0
		+ 1 collider	1.80 (1.31, 2.48)	15.2	-0.10	-14.9	0.04	0.62	89.2	95.7
		All covariates	1.68 (1.20, 2.35)	16.0	-0.17	-25.2	0.06	0.64	80.5	87.8
	Monte Carlo SE (min~max)				0.0044~ 0.0054	0.63~0.78	0.0011~ 0.0031	0.0003~ 0.0004	0.01~ 0.02	0~0.01
Ref	$N = 20$	No covariates	2.82 (2.46, 3.22)	7.5	0.34	49.4	0.12	0.27	0.1	100.0
	$OR_{AY} = 2$	1 confounder	2.68 (2.34, 3.07)	7.8	0.29	42.4	0.09	0.27	1.7	100.0
	($\beta = 0.7$)	2 confounders	2.53 (2.21, 2.91)	8.1	0.24	34.1	0.06	0.28	7.4	100.0
		3 confounders	2.41 (2.09, 2.78)	8.1	0.19	27.0	0.04	0.28	25.5	100.0
		4 confounders	2.28 (1.98, 2.63)	8.3	0.13	19.0	0.02	0.29	57.3	100.0
		5 confounders	2.16 (1.87, 2.50)	8.5	0.08	11.4	0.01	0.29	81.5	100.0
		6 confounders	2.01 (1.74, 2.34)	8.8	0.01	1.0	0.01	0.30	94.0	100.0
		+ 1 risk factor	2.05 (1.76, 2.38)	9.3	0.02	3.4	0.01	0.30	93.5	100.0
		+ 1 mediator	1.86 (1.59, 2.16)	8.9	-0.07	-10.8	0.01	0.31	83.7	100.0
		+ 1 collider	1.81 (1.55, 2.11)	9.0	-0.10	-14.7	0.02	0.31	75.3	100.0
		All covariates	1.68 (1.44, 1.98)	9.5	-0.17	-24.8	0.04	0.33	42.9	100.0
	Monte Carlo SE (min~max)				0.0022~ 0.0026	0.31~0.37	0.0003~ 0.0015	0.0001~ 0.0001	0.00~ 0.02	0~0

7-2	$N = 50$	No covariates	2.82 (2.59, 3.06)	5.2	0.34	49.4	0.12	0.17	0	100.0
	$OR_{AY} = 2$	1 confounder	2.69 (2.47, 2.92)	5.5	0.29	42.5	0.09	0.17	0	100.0
	($\beta = 0.7$)	2 confounders	2.53 (2.33, 2.76)	5.6	0.24	34.2	0.06	0.18	0	100.0
		3 confounders	2.41 (2.21, 2.64)	5.8	0.19	27.0	0.04	0.18	1.3	100.0
		4 confounders	2.28 (2.09, 2.50)	6.0	0.13	19.1	0.02	0.18	18.9	100.0
		5 confounders	2.17 (1.98, 2.37)	6.4	0.08	11.5	0.01	0.19	61.3	100.0
		6 confounders	2.02 (1.83, 2.22)	6.5	0.01	1.1	0.00	0.19	94.9	100.0
		+ 1 risk factor	2.05 (1.86, 2.26)	6.8	0.02	3.6	0.00	0.19	91.6	100.0
		+ 1 mediator	1.86 (1.69, 2.05)	6.9	-0.07	-10.6	0.01	0.19	68.6	100.0
		+ 1 collider	1.81 (1.64, 2.00)	6.6	-0.10	-14.5	0.01	0.19	47.0	100.0
		All covariates	1.69 (1.52, 1.87)	7.0	-0.17	-24.6	0.03	0.20	8.7	100.0
	Monte Carlo SE (min~max)				0.0013~ 0.0016	0.19~0.24	0.0001~ 0.0009	0.0000~ 0.0000	0~0.02	0~0

OR, odds ratio; CI, confidence interval; MSE, mean square error; *A*, exposure; *Y*, outcome; SE, standard error.
Scales of meta-analyses had little impact on the precision of pooled ORs. Only when the number of included studies was small, heterogeneity among studies might show up and led to less stability in effect estimations.

Table S10. Performances of statistical adjustment strategies in scenarios with different pooling methods in meta-analyses

Scenario	Description	Adjustment strategy	Pooled $\widehat{OR}$ (95% $\widehat{CI}$)	I^2 (%)	Performance of $\hat{\beta} = \ln(\widehat{OR})$					
					Bias	Relative bias (%)	MSE	Width of CI	Coverage (%)	Power (%)
Ref	Same source: fixed-effect model $OR_{AY} = 2$ ($\beta = 0.7$)	No covariates	2.82 (2.46, 3.22)	7.5	0.34	49.4	0.12	0.27	0.1	100.0
		1 confounder	2.68 (2.34, 3.07)	7.8	0.29	42.4	0.09	0.27	1.7	100.0
		2 confounders	2.53 (2.21, 2.91)	8.1	0.24	34.1	0.06	0.28	7.4	100.0
		3 confounders	2.41 (2.09, 2.78)	8.1	0.19	27.0	0.04	0.28	25.5	100.0
		4 confounders	2.28 (1.98, 2.63)	8.3	0.13	19.0	0.02	0.29	57.3	100.0
		5 confounders	2.16 (1.87, 2.50)	8.5	0.08	11.4	0.01	0.29	81.5	100.0
		6 confounders	2.01 (1.74, 2.34)	8.8	0.01	1.0	0.01	0.30	94.0	100.0
		+ 1 risk factor	2.05 (1.76, 2.38)	9.3	0.02	3.4	0.01	0.30	93.5	100.0
		+ 1 mediator	1.86 (1.59, 2.16)	8.9	-0.07	-10.8	0.01	0.31	83.7	100.0
		+ 1 collider	1.81 (1.55, 2.11)	9.0	-0.10	-14.7	0.02	0.31	75.3	100.0
		All covariates	1.68 (1.44, 1.98)	9.5	-0.17	-24.8	0.04	0.33	42.9	100.0
	Monte Carlo SE (min~max)				0.0022~ 0.0026	0.31~0.37	0.0003~ 0.0015	0.0001~ 0.0001	0.00~ 0.02	0~0
8-1	Different sources: fixed-effect model $OR_{AY} = 2$ ($\beta = 0.7$)	No covariates	2.44 (1.91, 3.14)	68.2	0.20	29.0	0.06	0.26	27.7	100.0
		1 confounder	2.29 (1.82, 2.88)	55.7	0.14	19.7	0.03	0.27	45.9	100.0
		2 confounders	2.19 (1.77, 2.72)	47.5	0.09	13.3	0.02	0.27	62.7	100.0
		3 confounders	2.15 (1.75, 2.65)	41.5	0.07	10.7	0.02	0.28	68.6	100.0
		4 confounders	2.10 (1.71, 2.56)	36.3	0.05	6.9	0.01	0.28	79.0	100.0
		5 confounders	2.03 (1.67, 2.48)	31.8	0.02	2.5	0.01	0.29	81.9	100.0
		6 confounders	2.01 (1.66, 2.44)	30.4	0.00	0.7	0.01	0.29	85.8	100.0
		+ 1 risk factor	2.04 (1.66, 2.52)	30.3	0.02	3.1	0.01	0.30	82.8	100.0
		+ 1 mediator	1.94 (1.58, 2.36)	32.8	-0.03	-4.6	0.02	0.30	81.8	100.0
		+ 1 collider	1.90 (1.54, 2.33)	31.5	-0.05	-7.6	0.01	0.30	79.4	100.0
		All covariates	1.84 (1.47, 2.32)	36.5	-0.08	-11.8	0.02	0.31	70.9	100.0
	Monte Carlo SE (min~max)				0.0031~ 0.0040	0.45~0.58	0.0004~ 0.0017	0.0008~ 0.0011	0.01~ 0.02	0~0

8-2	Different	No covariates	2.39 (1.85, 3.09)	68.2	0.18	25.5	0.05	0.50	69.0	100.0
	sources:	1 confounder	2.24 (1.81, 2.77)	55.7	0.11	16.2	0.02	0.44	80.3	100.0
	random-	2 confounders	2.15 (1.75, 2.65)	47.5	0.07	10.6	0.02	0.41	85.8	100.0
	effects	3 confounders	2.13 (1.74, 2.60)	41.5	0.06	8.8	0.01	0.40	87.8	100.0
	model	4 confounders	2.08 (1.72, 2.51)	36.3	0.04	5.5	0.01	0.38	92.4	100.0
	$OR_{AY} = 2$	5 confounders	2.04 (1.69, 2.46)	31.8	0.02	2.6	0.01	0.38	93.6	100.0
	($\beta = 0.7$)	6 confounders	2.02 (1.68, 2.43)	30.4	0.01	1.3	0.01	0.38	94.7	100.0
		+ 1 risk factor	2.06 (1.69, 2.51)	30.3	0.03	4.2	0.01	0.39	92.0	100.0
		+ 1 mediator	1.97 (1.62, 2.40)	32.8	-0.02	-2.4	0.01	0.40	94.1	100.0
		+ 1 collider	1.92 (1.59, 2.34)	31.5	-0.04	-5.8	0.01	0.39	91.4	100.0
		All covariates	1.89 (1.51, 2.35)	36.5	-0.06	-8.5	0.02	0.43	89.4	100.0
	Monte Carlo SE				0.0030~	0.43~0.60	0.0004~	0.0021~	0.01~	0~0
	(min~max)				0.0041		0.0016	0.0027	0.01	
8-3	Different	No covariates	2.39 (1.85, 3.09)	68.2	0.18	25.5	0.05	0.50	68.9	100.0
	sources:	1 confounder	2.24 (1.81, 2.77)	55.7	0.11	16.2	0.02	0.43	79.4	100.0
	proper	2 confounders	2.15 (1.75, 2.65)	47.5	0.07	10.6	0.02	0.40	84.9	100.0
	model based	3 confounders	2.13 (1.74, 2.60)	41.5	0.06	8.9	0.01	0.39	85.7	100.0
	on Q test	4 confounders	2.08 (1.72, 2.52)	36.3	0.04	5.6	0.01	0.37	89.7	100.0
	$OR_{AY} = 2$	5 confounders	2.04 (1.68, 2.46)	31.8	0.02	2.6	0.01	0.36	92.1	100.0
	($\beta = 0.7$)	6 confounders	2.02 (1.67, 2.43)	30.4	0.01	1.3	0.01	0.36	92.8	100.0
		+ 1 risk factor	2.06 (1.69, 2.51)	30.3	0.03	4.0	0.01	0.37	90.5	100.0
		+ 1 mediator	1.96 (1.61, 2.40)	32.8	-0.02	-2.6	0.01	0.38	91.5	100.0
		+ 1 collider	1.92 (1.57, 2.34)	31.5	-0.04	-6.0	0.01	0.38	89.3	100.0
		All covariates	1.88 (1.51, 2.35)	36.5	-0.06	-8.7	0.02	0.42	86.6	100.0
	Monte Carlo SE				0.0030~	0.44~0.60	0.0004~	0.0024~	0.01~	0~0
	(min~max)				0.0041		0.0016	0.0028	0.01	

OR, odds ratio; CI, confidence interval; MSE, mean square error; *A*, exposure; *Y*, outcome; SE, standard error.

If some included studies were obtained from different sources, random-effects model presented significantly better performance than fixed-effects model. However, neither the source of original studies nor the model used in meta-analyses affected the accuracy of full adjustment strategy.

Table S11. Case-control studies included in an empirical meta-analysis on passive smoking and breast cancer [7]

Study	Number of cases	Selection of controls	Matching approach*	Source of exposure	Original OR (95% CI)	Adjusted covariates	Primary analysis‡
Li 2015 [8]	877	Hospital	Frequency	Cohabitant	1.40 (1.15, 1.71)†	**Age**, **residence**, study stage, **BMI**, **physical activity**, age at menarche, age at first live birth, age at menopause, **family history of breast cancer**, <u>history of benign breast disease</u>	No (↓)
Tong 2014 [9]	312	Hospital	Frequency	Spouse	1.46 (1.05, 2.03)	**Age**, age at menarche, menopausal status, oral contraceptive use, **family history of cancer**, **alcohol**, **BMI**	Yes
Nishino 2014 [10]	773	Hospital	Frequency	Spouse	1.09 (0.91, 1.31)†	**Age**, **BMI**, **occupation**, **physical activity**, menopausal status, age at menarche, age at menopause, age at first birth, **family history of breast cancer**, parity, use of hormones (oral contraceptive/HRT), referral status, year of recruitment, **area of residence**, **alcohol**, hormone receptor status	Yes
Tang 2013 [11]	765	Hospital	Frequency	Cohabitant	1.55 (1.23, 1.96)	**Age**, **marital status**, **physical activity**, **alcohol**, age at menarche, menopausal status, **BMI**, parity, **education**, **family history of breast cancer**	Yes
Ilic 2013 [5]	130	Hospital	Frequency	Home or work	1.57 (0.81, 3.03)	**Education**, **marital status**, age at menarche, menopausal status, breastfeeding history, **family history of breast cancer**, **BMI**, **alcohol**, <u>cardiovascular disease</u> **(age was not included due to collinearity)**	No (↓)

Anderson 2012 [12]	918	Population	Frequency	Cohabitant	1.08 (0.89, 1.31)†	**Age**, **physical activity** (premenopausal women only)	No (↑)
De Silva 2010 [13]	100	Population	Frequency	Any	2.96 (1.53, 5.75)	Lifetime duration of breastfeeding, age at first pregnancy, menopausal status, previous abortions, **education**, **employment status**, **family history of breast cancer**	No (↑)
Conlon 2010 [14]	129	Population	Frequency	Home or work	1.15 (0.61, 2.18)†	None	No (↑)
Chilian-Herrera 2010	504	Population	Unknown	Home or work	3.34 (2.38, 4.68)	Reproductive breast cancer risk factors (unspecified)	No (↑)
Ahern 2009 [15]	232	Population	Frequency	Home or work	0.86 (0.57, 1.31)†	**Age**, menopausal status, **BMI**, parity, **alcohol**, **family history of breast cancer**	Yes
Slattery 2008 [6]	1347	Population	Frequency	Any	1.05 (0.88, 1.27)†	**Age**, **center**, **BMI**, aspirin/NSAID use, parity, **alcohol**, **physical activity**, menopausal status, **ethnicity**, hormone use (postmenopausal women only)	No (↓)
Rollison 2008 [16]	124	Population	Frequency	Cohabitant	0.98 (0.58, 1.64)	**Age**, menopausal status, **BMI**, age at menarche, age at first live birth, oral contraceptive use, other hormone use, **family history of breast cancer**, **alcohol**	Yes
Roddam 2007 [17]	297	General practitioner	Individually (1:1)	Spouse	0.89 (0.64, 1.25)	Matched variables: **age**, **region**; Adjusted variables: **socioeconomic status**, **alcohol**, **BMI**, parity, **family history of breast cancer**, use of contraceptives, age at menarche, menopausal status	Yes
Mechanic 2006 [18]	1211	Population	Frequency	Cohabitant	1.10 (0.93, 1.31)†	**Age**, **race**, offsets, age at menarche, age at first full-term pregnancy/parity composite, **family history**, **alcohol (income, education, and BMI were not included**	Yes

						**due to unchanged results)**	
Lissowska 2006 [19]	1034	Population	Frequency	Cohabitant	0.92 (0.74, 1.14)†	**Age**, **site**, **education**, age at menarche, number of full-term births, age at first full-term birth, age at menopause, **BMI**, **family history of breast cancer**, history of benign breast biopsy, previous screening mammography, contraceptive/HRT use	No (↓)
Sillanpaa 2005 [20]	363	Population	Frequency	Home or work	0.85 (0.62, 1.16)	**Age**, age at menarche, age at first full-term pregnancy, number of pregnancies, **family history of breast cancer**, history of benign breast diseases, **alcohol (BMI was examined by stratified analysis)**	No (↓)
Bonner 2005 [21]	525	Population	Frequency	Cohabitant	1.18 (0.86, 1.63)†	**Age**, **education**, **race**, previous benign breast disease, parity, age at menarche, **BMI**, age at first birth, **family history of breast cancer**, **alcohol**, age at menopause, menopausal status	No (↓)
Shrubsole 2004 [22]	813	Population	Frequency	Spouse	1.00 (0.80, 1.20)	**Age**, **education**, **family history of breast cancer**, history of fibroadenoma, age at menarche, parity, age at first birth, menopausal status, age at menopause, **physical activity**, **waist-to-hip ratio**	No (↓)
Gammon 2004 [23]	598	Population	Frequency	Cohabitant	1.04 (0.81, 1.35)	**Age**, history of benign breast disease, **BMI at age 20**, **family history of breast cancer**, fertility problems, number of pregnancies, menopausal status, **weight in year before reference date (lifestyle behaviors, race, ethnicity, education, religion, and marital status were not included due to unchanged results)**	No (↓)

Lash 2002 [3]	305	Population	Individually (1:1)	Cohabitant	0.85 (0.63, 1.10)	Matched variables: **age**, vital status; Adjusted variables: history of radiation therapy, **BMI**, **family history of breast cancer**, history of breast cancer, history of benign breast disease, **alcohol**, parity, age at first birth	No (↓)
Kropp 2002 [24]	197	Population	Individually (1:2)	Home or work	1.69 (1.16, 2.45)†	Matched variables: **age**, **study region**; Adjusted variables: **alcohol**, breastfeeding, **education**, **family history of breast cancer**, menopausal status, **BMI**	Yes
Rookus 2000	918	Population	Unknown	Home or work	1.20 (0.80, 1.70)	**Lifetime physical activity**, other confounders (unspecified)	No (↑)
Liu 2000 [25]	186	Hospital	Individually (1:1)	Cohabitant	1.49 (0.96, 2.30)†	Matched variables: **age at diagnosis**, date of diagnosis, **marital status**	No (↑)
Johnson 2000 [26]	606	Population	Frequency	Home or work	1.47 (1.06, 2.04)†	**Age**, **province**, **education**, **BMI**, **alcohol**, **physical activity**, age at menarche, age at end of first pregnancy, number of live births, months of breastfeeding, **height**, menopausal status	No (↓)
Delfino 2000 [27]	64	Benign breast disease	Frequency	Cohabitant	1.50 (0.79, 2.87)	**Age**, menopausal status, **family history of breast cancer**	No (↑)
Lash 1999 [4]	120	Population	Frequency	Cohabitant	2.00 (1.10, 3.70)	**Age**, **BMI**, parity, history of radiation therapy, **family history of breast cancer**, history of breast cancer, history of benign breast disease, **alcohol**	No (↓)
Morabia 1996 [28]	90	Population	Frequency	Spouse	3.10 (1.60, 6.10)	**Age**, **education**, **BMI**, age at menarche, age at first live birth, oral contraception, **family history of breast cancer**, history of breast biopsy, **saturated fat**, **alcohol**	No (↓)

Smith 1994 [29]	94	Population	Frequency	Spouse	1.58 (0.81, 3.10)	**Age**, **region**, age at menarche, nulliparity, age at first full-term pregnancy, breast feeding, oral contraceptive use, **family history of breast cancer**, <u>biopsy for benign breast disease</u>, **alcohol**	No (↑)
Sandler 1985 [30]	32	Friends	Unknown	Spouse	1.62 (0.76, 3.44)	**Age**	No (↑)

OR, odds ratio; CI, confidence interval; BMI, body mass index; HRT, hormone replacement therapy; NSAID, nonsteroidal anti-inflammatory drug.

* Decided by the model used for calculating ORs (unconditional logistic regression model: frequency matching; condition logistic regression model: individually matching).

† Estimated by Lee and Hamling from data reported in original case-control studies.

‡ "↑" means overestimation, and "↓" means underestimation.

Among 29 case-control studies included in the meta-analysis, 8 (27.6%) gave reasonable effect estimations and were included in the primary analysis; 12 (41.4%) were underestimated due to not adjusting for negative confounders of family history (2/12), adjusting for mediators of benign breast disease (9/12), or adjusting for colliders of cardiovascular disease (1/12); 9 (31.0%) were overestimated due to not adjusting for positive confounders of age or body mass index.

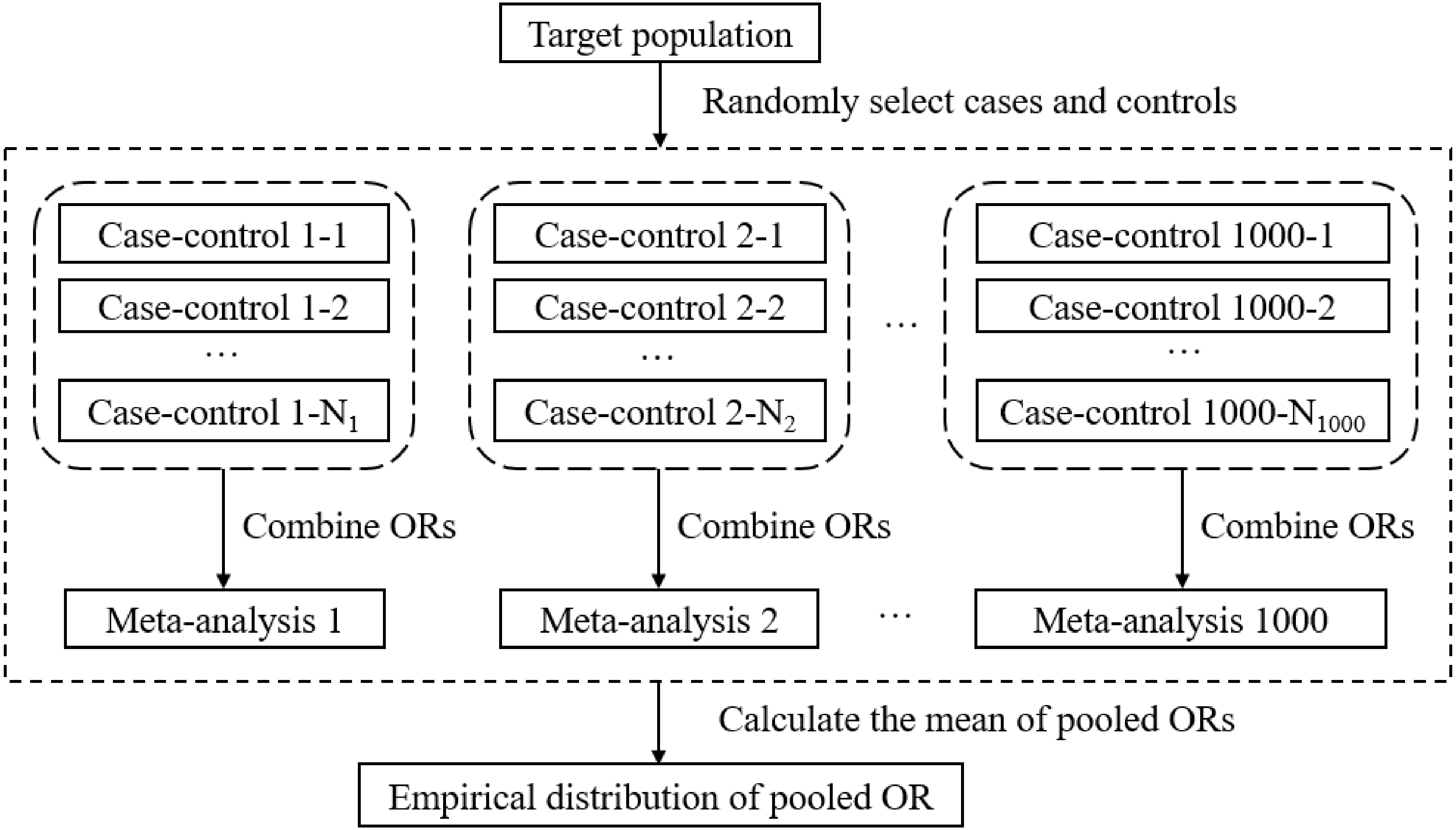

Figure S1. Flow chart of the simulation study

OR, odds ratio.

A target population was simulated with pre-determined exposure, outcome, and covariates. Then case-control studies were randomly selected from the population, and a series of original ORs were calculated in each case-control study by different adjustment strategies. Then meta-analyses were conducted to pool these ORs. The process was repeated for 1000 times to obtain the empirical distribution of pooled OR.

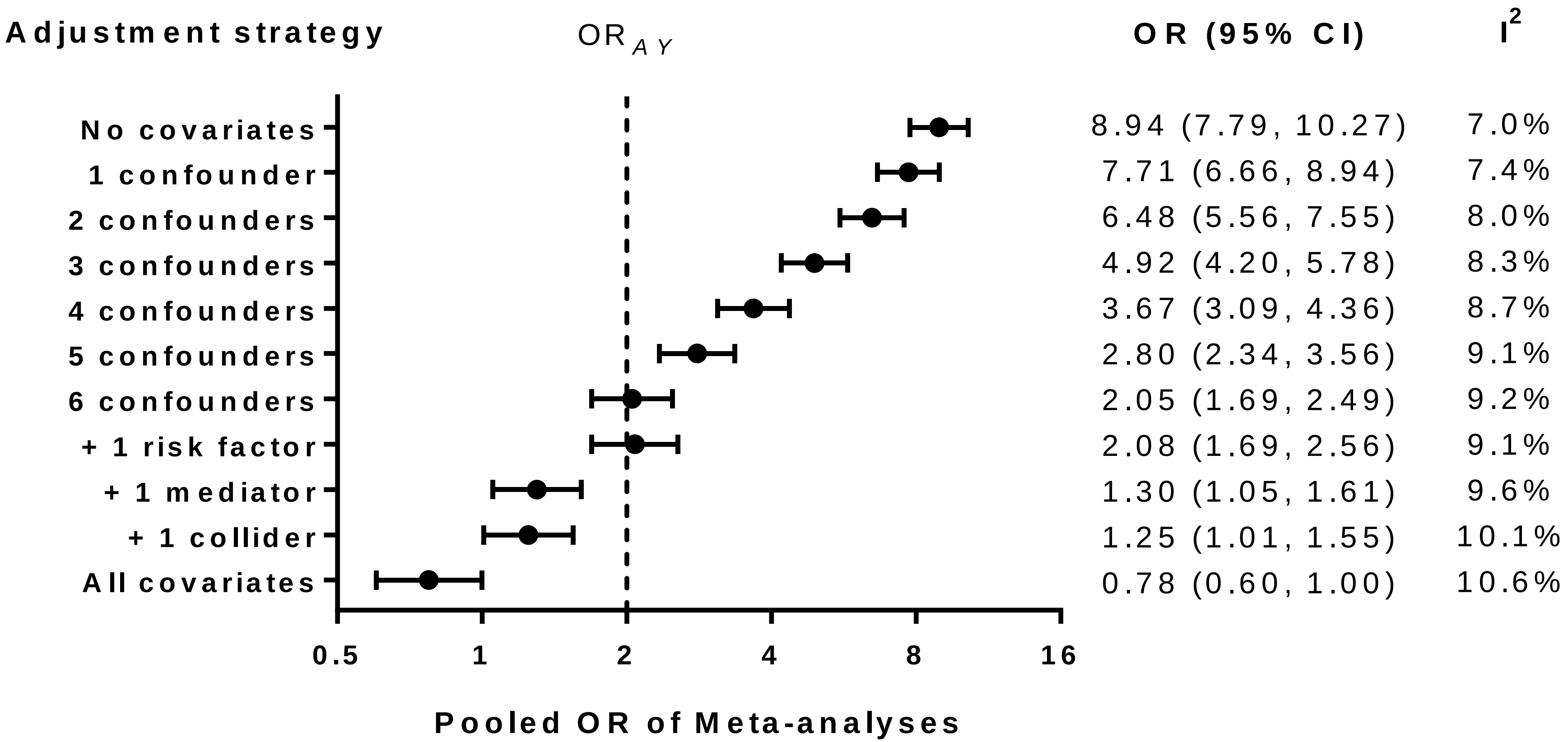

Figure S2. Pooled ORs of meta-analyses in scenario Ref' where all covariates were continuous and shared the same inter-variable relationships with scenario Ref (OR_{AY} = 2)

OR, odds ratio; CI, confidence interval; *A*, exposure; *Y*, outcome.

If the covariates $\boldsymbol{U} = [\boldsymbol{L}, R, M, C]$ were continuous rather than categorical, the pooled ORs showed more sensitivity to the adjustment strategies.

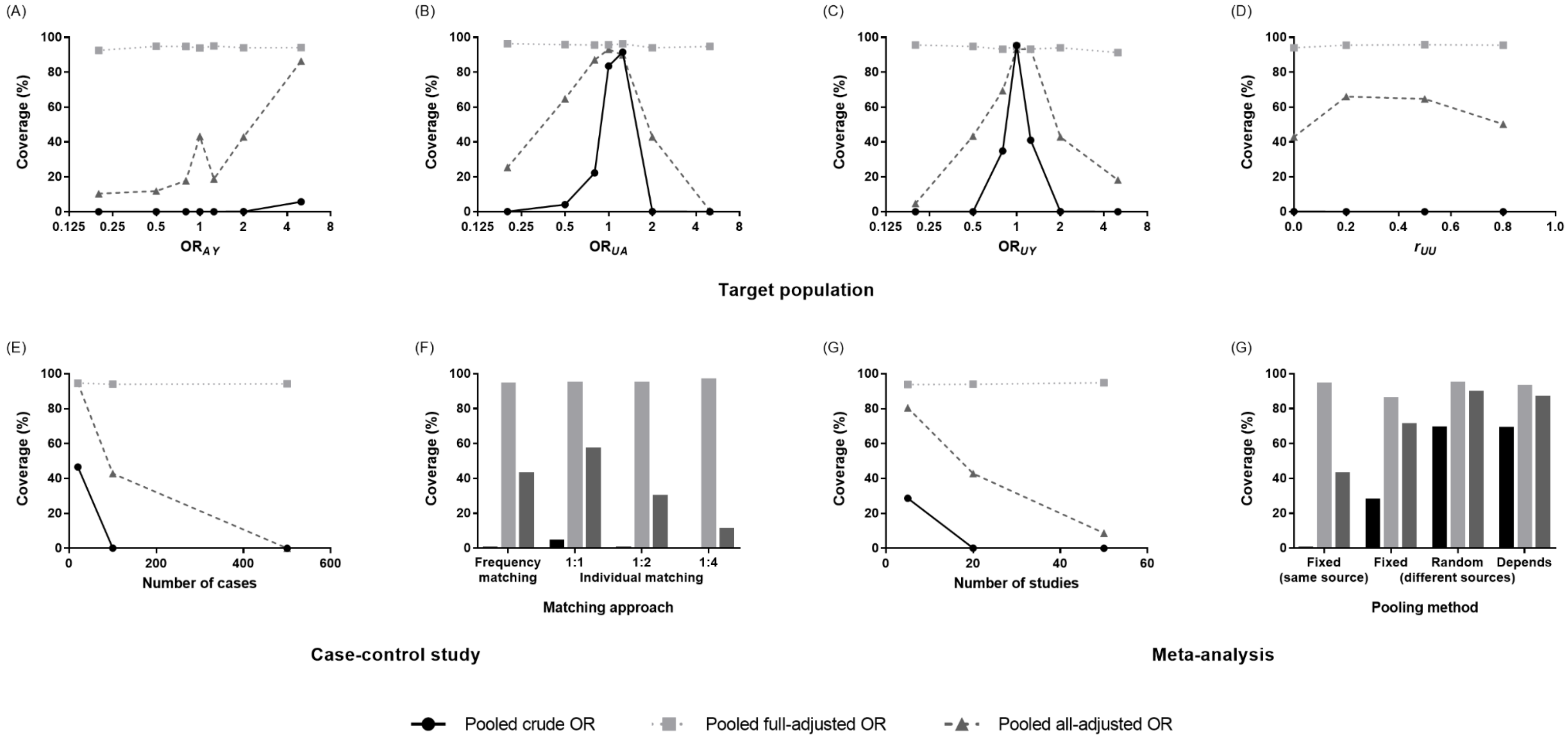

Figure S3. Coverage of effect estimations under different adjustment strategies

Coverage was presented according to different (A) OR_{AY}, (B) OR_{UA}, (C) OR_{UY}, and (D) r_{UU} in the target population; (E) number of cases and (F) matching approach in original the case-control studies; and (G) number of studies and (H) pooling method in the meta-analyses.

OR, odds ratio; *A*, exposure; *Y*, outcome; ***U***, covariate.

For all scenarios, pooled full-adjusted ORs showed the best coverage. With insufficient or improper adjustment of covariates in original studies, the confidence interval of pooled effect estimation had unacceptable coverage possibilities.

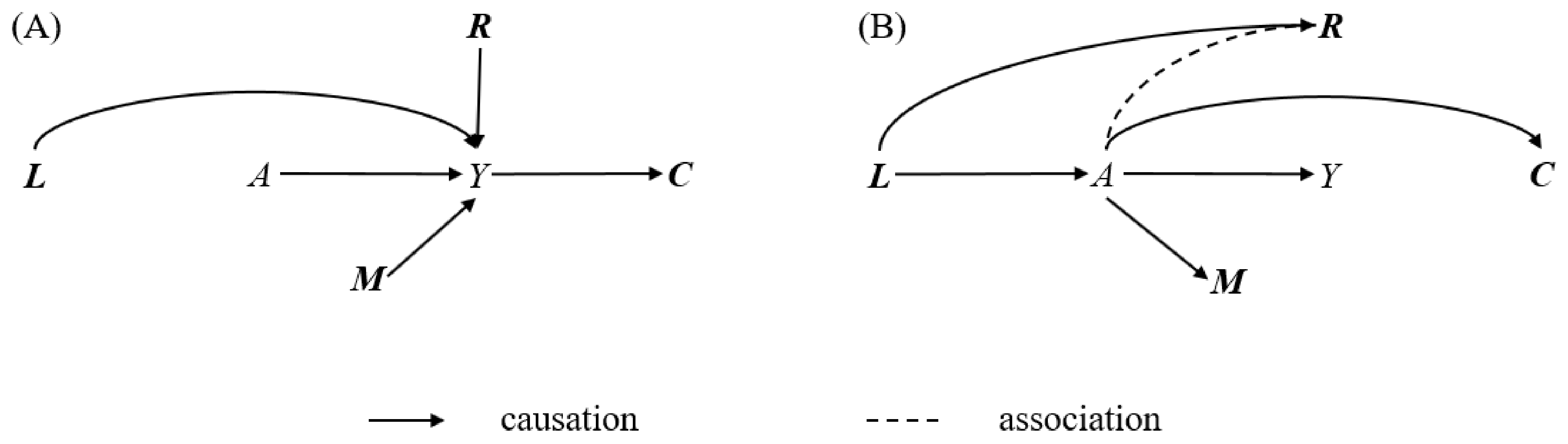

Figure S4. Directed acyclic graph in the target population of (A) scenario 2-4 [OR_{UA} = 1] and (B) scenario 3-4 [OR_{UY} = 1]
A, exposure; *Y*, outcome; ***U***, covariate; ***L***, confounder; ***R***, risk factor; ***M***, mediator; ***C***, collider.
If the covariates ***U*** = [***L***, *R*, *M*, *C*] had no associations with exposure *A* (A) or outcome *Y* (B), they might not be recognized as confounders in original studies. Whether adjusted for these variables would not affect the estimation of causal effect.

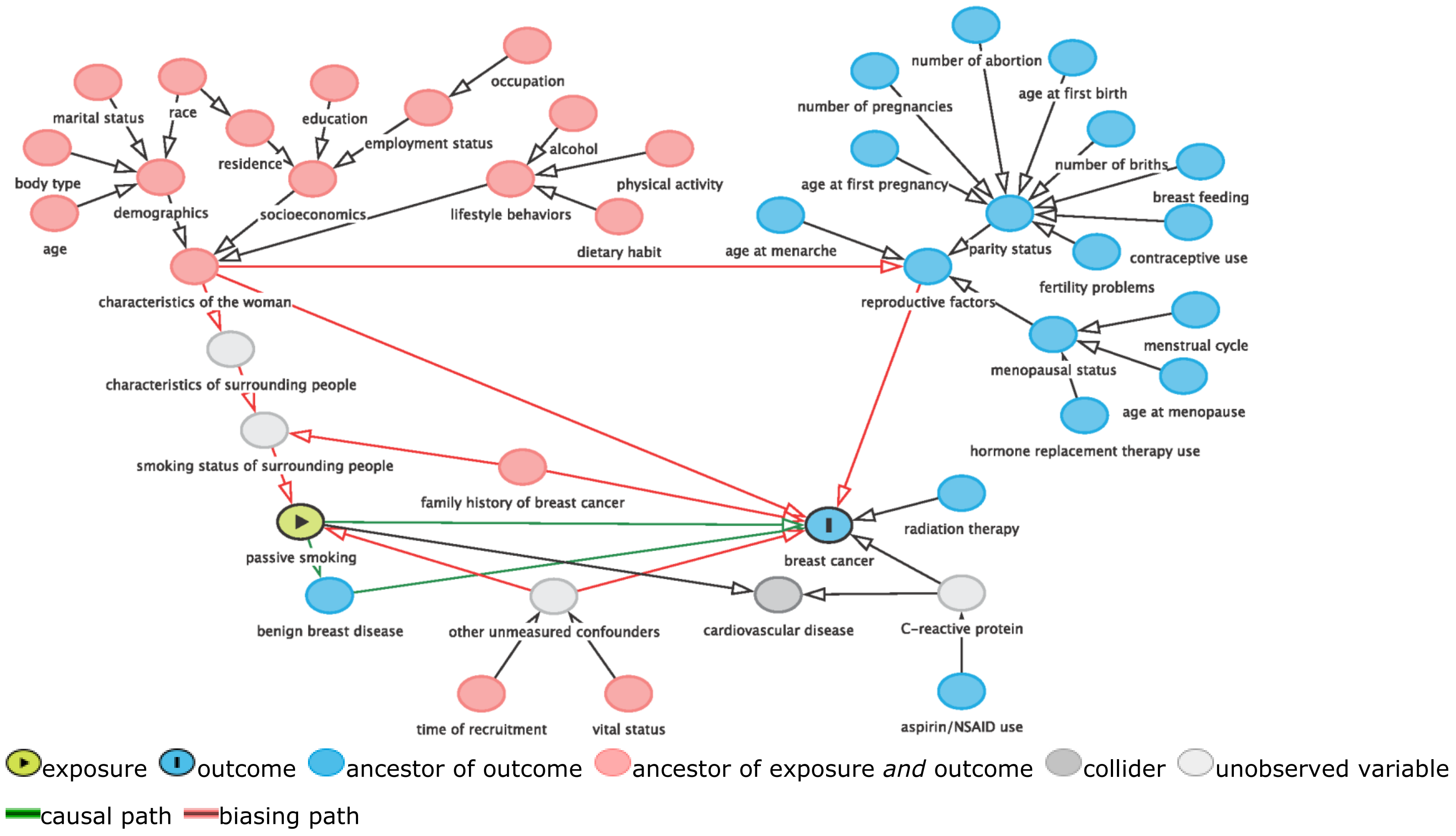

Figure S5. Directed acyclic graph between passive smoking and breast cancer

NSAID, nonsteroidal anti-inflammatory drug.

In the causal path from passive smoking to breast cancer, women's characteristics and family history of breast cancer were considered to be confounders; reproductive factors, radiation therapy, and aspirin/NSAID use were considered to be risk factors; benign breast disease was considered to be a mediator; cardiovascular disease was considered to be a collider. For individual matched studies, cases and controls were also matched by time of recruitment and vital status to balance unmeasured confounders.

References

1. Emrich LJ, Piedmonte MR. A method for generating high-dimensional multivariate binary variates. Am Stat. 1991;45(4):302-4.

2. National Comprehensive Cancer Network. NCCN Clinical Practice Guidelines in Oncology (NCCN Guidelines): Breast Cancer Risk Reduction. 2019. Available from: https://www.nccn.org/professionals/physician_gls/pdf/breast_risk.pdf.

3. Lash TL, Aschengrau A. A null association between active or passive cigarette smoking and breast cancer risk. Breast Cancer Res Treat. 2002;75(2):181-4.

4. Lash TL, Aschengrau A. Active and passive cigarette smoking and the occurrence of breast cancer. Am J Epidemiol. 1999;149(1):5-12.

5. Ilic M, Vlajinac H, Marinkovic J. Cigarette smoking and breast cancer: a case-control study in Serbia. Asian Pac J Cancer Prev. 2014;14(11):6643-7.

6. Slattery ML, Curtin K, Giuliano AR, Sweeney C, Baumgartner R, Edwards S, et al. Active and passive smoking, IL6, ESR1, and breast cancer risk. Breast Cancer Res Treat. 2008;109(1):101-11.

7. Lee PN, Hamling JS. Environmental tobacco smoke exposure and risk of breast cancer in nonsmoking women. An updated review and meta-analysis. Inhal Toxicol. 2016;28(10):431-54.

8. Li B, Wang L, Lu MS, Mo XF, Lin FY, Ho SC, et al. Passive Smoking and Breast Cancer Risk among Non-Smoking Women: A Case-Control Study in China. PLoS One. 2015;10(4):e0125894.

9. Tong JH, Li Z, Shi J, Li HM, Wang Y, Fu LY, et al. Passive smoking exposure from partners as a risk factor for ER+/PR+ double positive breast cancer in never-smoking Chinese urban women: a hospital-based matched case control study. PLoS One. 2014;9(5):e97498.

10. Nishino Y, Minami Y, Kawai M, Fukamachi K, Sato I, Ohuchi N, et al. Cigarette smoking and

breast cancer risk in relation to joint estrogen and progesterone receptor status: a case-control study in Japan. Springerplus. 2014;3:65.

11. Tang LY, Chen LJ, Qi ML, Su Y, Su FX, Lin Y, et al. Effects of passive smoking on breast cancer risk in pre/post-menopausal women as modified by polymorphisms of PARP1 and ESR1. Gene. 2013;524(2):84-9.

12. Anderson LN, Cotterchio M, Mirea L, Ozcelik H, Kreiger N. Passive cigarette smoke exposure during various periods of life, genetic variants, and breast cancer risk among never smokers. Am J Epidemiol. 2012;175(4):289-301.

13. De Silva M, Senarath U, Gunatilake M, Lokuhetty D. Prolonged breastfeeding reduces risk of breast cancer in Sri Lankan women: a case-control study. Cancer Epidemiol. 2010;34(3):267-73.

14. Conlon MS, Johnson KC, Bewick MA, Lafrenie RM, Donner A. Smoking (active and passive), N-acetyltransferase 2, and risk of breast cancer. Cancer Epidemiol. 2010;34(2):142-9.

15. Ahern TP, Lash TL, Egan KM, Baron JA. Lifetime tobacco smoke exposure and breast cancer incidence. Cancer Causes Control. 2009;20(10):1837-44.

16. Rollison DE, Brownson RC, Hathcock HL, Newschaffer CJ. Case-control study of tobacco smoke exposure and breast cancer risk in Delaware. BMC Cancer. 2008;8:157.

17. Roddam AW, Pirie K, Pike MC, Chilvers C, Crossley B, Hermon C, et al. Active and passive smoking and the risk of breast cancer in women aged 36-45 years: a population based case-control study in the UK. Br J Cancer. 2007;97(3):434-9.

18. Mechanic LE, Millikan RC, Player J, de Cotret AR, Winkel S, Worley K, et al. Polymorphisms in nucleotide excision repair genes, smoking and breast cancer in African Americans and whites: a population-based case-control study. Carcinogenesis. 2006;27(7):1377-85.

19. Lissowska J, Brinton LA, Zatonski W, Blair A, Bardin-Mikolajczak A, Peplonska B, et al. Tobacco smoking, NAT2 acetylation genotype and breast cancer risk. Int J Cancer. 2006;119(8):1961-9.

20. Sillanpaa P, Hirvonen A, Kataja V, Eskelinen M, Kosma VM, Uusitupa M, et al. NAT2 slow acetylator genotype as an important modifier of breast cancer risk. Int J Cancer. 2005;114(4):579-84.

21. Bonner MR, Nie J, Han D, Vena JE, Rogerson P, Muti P, et al. Secondhand smoke exposure in early life and the risk of breast cancer among never smokers (United States). Cancer Causes Control. 2005;16(6):683-9.

22. Shrubsole MJ, Gao YT, Dai Q, Shu XO, Ruan ZX, Jin F, et al. Passive smoking and breast cancer risk among non-smoking Chinese women. Int J Cancer. 2004;110(4):605-9.

23. Gammon MD, Eng SM, Teitelbaum SL, Britton JA, Kabat GC, Hatch M, et al. Environmental tobacco smoke and breast cancer incidence. Environ Res. 2004;96(2):176-85.

24. Kropp S, Chang-Claude J. Active and passive smoking and risk of breast cancer by age 50 years among German women. Am J Epidemiol. 2002;156(7):616-26.

25. Liu L, Wu K, Lin X, Yin W, Zheng X, Tang X, et al. Passive Smoking and Other Factors at Different Periods of Life and Breast Cancer Risk in Chinese Women who have Never Smoked - A Case-control Study in Chongqing, People's Republic of China. Asian Pac J Cancer Prev. 2000;1(2):131-7.

26. Johnson KC, Hu J, Mao Y, Canadian Cancer Registries Epidemiology Research G. Passive and active smoking and breast cancer risk in Canada, 1994-97. Cancer Causes Control. 2000;11(3):211-21.

27. Delfino RJ, Smith C, West JG, Lin HJ, White E, Liao SY, et al. Breast cancer, passive and active

cigarette smoking and Nacetyltransferase 2 genotype. Pharmacogenetics. 2000;10(5):461-9.

28. Morabia A, Bernstein M, Heritier S, Khatchatrian N. Relation of breast cancer with passive and active exposure to tobacco smoke. Am J Epidemiol. 1996;143(9):918-28.

29. Smith SJ, Deacon JM, Chilvers CE. Alcohol, smoking, passive smoking and caffeine in relation to breast cancer risk in young women. UK National Case-Control Study Group. Br J Cancer. 1994;70(1):112-9.

30. Wells AJ. Re: "Breast cancer, cigarette smoking, and passive smoking". Am J Epidemiol. 1998;147(10):991-2.
