# Supplementary material for: Can statistical adjustment guided by causal inference improve the accuracy of effect estimation? A simulation and empirical research based on meta-analyses of case–control studies
Source: BMC Med Inform Decis Mak. 2020 Dec 11;20:333. doi: 10.1186/s12911-020-01343-3 (PMC7731571; doi:10.1186/s12911-020-01343-3)
Supplement: Supplementary file 2 — Additional file 2: Main SAS code of the simulation for scenario Ref. [file 12911_2020_1343_MOESM2_ESM.pdf]

Can statistical adjustment guided by causal inference improve the accuracy of effect estimation? A simulation and empirical research based on meta-analyses of case-control studies

Additional file 2. Main SAS code of the simulation for scenario Ref

Ruohua Yan[1], Tianyi Liu[1,2], Yaguang Peng[1], Xiaoxia Peng[1*]

1. Center for Clinical Epidemiology and Evidence-based Medicine, Beijing Children's Hospital, Capital Medical University, National Center for Children's Health, Nanlishilu 56, Xicheng District, Beijing 100045, China

2. Evidence Generation, Medical Affairs, AstraZenaca, Level 22, International Fortune Center, Jianguomenwai Avenue 8, Chaoyang District, Beijing 100010, China

* Correspondence: pengxiaoxia@bch.com.cn

1. Generation of target population

```
*1.1 Simulating confounders L1-L6;
%include 'D:\Simulation\Programs\RandMVBinary.sas'; /*Wicklin R. Simulating
Data with SAS. Cary, NC: SAS Institute Inc; 2013.*/

%let id=Ref;
ods output L=population;
proc iml;
	load module=_all_;
	p = {0.2 0.2 0.2 0.2 0.2 0.2}; /*positive probability*/
	R = {1 0 0 0 0 0, 0 1 0 0 0 0, 0 0 1 0 0 0, 0 0 0 1 0 0, 0 0 0 0 1 0, 0 0 0 0 0 1};
/*correlation matrix*/
	L = RandMVBinary(10000, p, R);
	DiffMean = p - mean(L);
	DiffCorr = R - corr(L);
	call svd(U,Q,V,R);
	print L;
quit;

data population;
	set population;
	rename col1=L1 col2=L2 col3=L3 col4=L4 col5=L5 col6=L6;
run;

*1.2 Simulating exposure A, risk factor R, mediator M, outcome Y, and collider C;
data pop;
	set population;
	lnA=log(0.2/0.8)+log(2)*L1+log(2)*L2+log(2)*L3+log(2)*L4+log(2)*L5+log(2
)*L6;
	pA=exp(lnA)/(1+exp(lnA));
	A=rand('bernoulli',pA);

	lnR=log(0.2/0.8)+log(2)*L1+log(2)*L2+log(2)*L3+log(2)*L4;
	pR=exp(lnR)/(1+exp(lnR));
	R=rand('bernoulli',pR);

	lnM=log(0.2/0.8)+log(2)*A;
	pM=exp(lnM)/(1+exp(lnM));
	M=rand('bernoulli',pM);
run;

data pop;
	set pop;
```

```
    lnY=log(0.2/0.8)
+log(2)*L1+log(2)*L2+log(2)*L3+log(2)*L4+log(2)*L5+log(2)*L6+(log(2)-
&pe*log(2))*A+log(2)*M+log(2)*R;
    pY=exp(lnY)/(1+exp(lnY));
    Y=rand('bernoulli',pY);

    lnC=log(0.2/0.8)+log(2)*A+log(2)*Y;
    pC=exp(lnC)/(1+exp(lnC));
    C=rand('bernoulli',pC);
run;

proc sort data=pop;
    by Y;
run;

proc export data=pop outfile="D:\Simulation\Results\population &id..csv" replace;
run;
```

2. Generation of case-control studies

```
*2.1 Sampling cases and controls;
%macro sample(repeat=, data=, nstudy=);
%do s=1 %to &nstudy;
     proc surveyselect data=&data method=srs n=100 out=sample_&repeat._&s;
        strata Y;
    run;
%end;
%mend sample;

*2.2 Estimating ORs;
%macro sampleOR(repeat=, nstudy=);
%do s=1 %to &nstudy;

    /*Crude OR*/
    ods output RelativeRisks=sampleOR_&repeat._&s._Crude;
    proc freq data=sample_&repeat._&s;
        tables A*Y /relrisk;
    run;

    data sampleOR_&repeat._&s._Crude;
        length type $20.;
        set sampleOR_&repeat._&s._Crude;
        if statistic='Odds Ratio';
        type='Crude';
```

```
        rename Value=sampleOR;
        keep Value LowerCL UpperCL type;
run;

/*ORs insufficiently adjusted for confounders*/
%do nvar=1 %to 6;

        ods output OddsRatios=sampleOR_&repeat._&s._Adjust&nvar;
        proc logistic data=sample_&repeat._&s;
                model Y(event='1')=A L1-L&nvar;
        run;

        data sampleOR_&repeat._&s._Adjust&nvar;
                length type $20.;
                set sampleOR_&repeat._&s._Adjust&nvar;
                if effect='A';
                type="Adjust&nvar";
                rename OddsRatioEst=sampleOR;
                keep OddsRatioEst LowerCL UpperCL type;
        run;

%end;

/*ORs improperly adjusted for risk factor, mediator or collider*/
ods output OddsRatios=sampleOR_&repeat._&s._Risk;
proc logistic data=sample_&repeat._&s;
        model Y(event='1')=A L1-L6 R;
run;

data sampleOR_&repeat._&s._Risk;
        length type $20.;
        set sampleOR_&repeat._&s._Risk;
        if effect='A';
        type="Risk";
        rename OddsRatioEst=sampleOR;
        keep OddsRatioEst LowerCL UpperCL type;
run;

ods output OddsRatios=sampleOR_&repeat._&s._Mediator;
proc logistic data=sample_&repeat._&s;
        model Y(event='1')=A L1-L6 M;
run;

data sampleOR_&repeat._&s._Mediator;
```

```
    length type $20.;
    set sampleOR_&repeat._&s._Mediator;
    if effect='A';
    type="Mediator";
    rename OddsRatioEst=sampleOR;
    keep OddsRatioEst LowerCL UpperCL type;
run;

ods output OddsRatios=sampleOR_&repeat._&s._Collider;
proc logistic data=sample_&repeat._&s;
    model Y(event='1')=A L1-L6 C;
run;

data sampleOR_&repeat._&s._Collider;
    length type $20.;
    set sampleOR_&repeat._&s._Collider;
    if effect='A';
    type="Collider";
    rename OddsRatioEst=sampleOR;
    keep OddsRatioEst LowerCL UpperCL type;
run;

ods output OddsRatios=sampleOR_&repeat._&s._All;
proc logistic data=sample_&repeat._&s;
    model Y(event='1')=A L1-L6 R M C;
run;

data sampleOR_&repeat._&s._All;
    length type $20.;
    set sampleOR_&repeat._&s._All;
    if effect='A';
    type="All";
    rename OddsRatioEst=sampleOR;
    keep OddsRatioEst LowerCL UpperCL type;
run;

/*All ORs*/
data sampleOR_&repeat._&s;
    length study $20.;
    set sampleOR_&repeat._&s._:;
    es=log(sampleOR); se=(log(sampleOR)-log(LowerCL))/1.96;
    study="s_&repeat._&s"; repeat=&repeat; nstudy=&s;
run;
```

```
%end;

data sampleOR_&repeat;
	set sampleOR_&repeat._1-sampleOR_&repeat._&nstudy;
run;

proc datasets lib=work;
	delete sample_&repeat._: sampleOR_&repeat._:;
run;
%mend sampleOR;
```

3. Generation of Meta-analyses

```
options mautosource sasautos=(' D:\Simulation\Programs');

%macro metaOR(repeat=, nstudy=, type=);
data sampleOR;
	set sampleOR_&repeat;
	if type="&type";
	w=1/(se**2);
run;

%mainverse(dataset=sampleOR,sampsize=no); /*Senn S, Weir J, Hua TA, et al.
Creating a suite of macros for meta-analysis in SAS: A case study in collaboration.
Stat Probabil Lett. 2011; 81 (7): 842-857.*/

data metaOR_&repeat._&type;
	length type $20.;
	set inv_var;
	if type='Combined';
	metaOR=exp(es); lowerCL=exp(lower); upperCL=exp(upper);
	repeat=&repeat; type="&type";
run;

proc sql; create table q as select es, se, w, sum(w) as denominator, sum(w*es) as
numerator from sampleOR; run;
data q; set q; e=w*((es-numerator/denominator)**2); run;
proc sql; create table i2 as select sum(e) as q from q; run;
data i2; set i2; i2=max(0,(q-&nstudy+1)/q); p=1-cdf('CHISQUARE',q,&nstudy-1);
run;

data metaOR_&repeat._&type;
	merge metaOR_&repeat._&type i2;
run;
```

```
%mend metaOR;
```

4. Repetition

```
%macro repeat(repeat=, data=, nstudy=, id=);
%do r=1 %to &repeat;

    %sample(repeat=&r,data=&data,nstudy=&nstudy);
    %sampleOR(repeat=&r,nstudy=&nstudy);
    %metaOR(repeat=&r,nstudy=&nstudy,type=Crude);
    %metaOR(repeat=&r,nstudy=&nstudy,type=Adjust1);
    %metaOR(repeat=&r,nstudy=&nstudy,type=Adjust2);
    %metaOR(repeat=&r,nstudy=&nstudy,type=Adjust3);
    %metaOR(repeat=&r,nstudy=&nstudy,type=Adjust4);
    %metaOR(repeat=&r,nstudy=&nstudy,type=Adjust5);
    %metaOR(repeat=&r,nstudy=&nstudy,type=Adjust6);
    %metaOR(repeat=&r,nstudy=&nstudy,type=Risk);
    %metaOR(repeat=&r,nstudy=&nstudy,type=Mediator);
    %metaOR(repeat=&r,nstudy=&nstudy,type=Collider);
    %metaOR(repeat=&r,nstudy=&nstudy,type=All);

    data metaOR_&r;
        set metaOR_&r._:;
    run;

    dm log 'clear' continue;
    dm odsresults 'clear' continue;

    proc datasets lib=work;
        delete metaOR_&r._:;
    run;

%end;

data metaOR;
    set metaOR_1-metaOR_&repeat;
run;

proc sort data=metaOR;
    by study;
run;

proc export data=metaOR outfile="D:\Simulation\Results\metaor &id..csv" replace;
run;
```

```
data sampleOR_full;
	length study $20.;
	set sampleOR_1-sampleOR_&repeat;
run;

proc export data=sampleOR_full outfile="D:\Simulation\Results\sampleor &id..csv"
replace;
run;
%mend repeat;

ods html close;
%repeat(repeat=1000,data=pop,nstudy=20,id=Ref);
ods html;
```
